# ToMExO: A probabilistic tree-structured model for cancer progression

**DOI:** 10.1371/journal.pcbi.1010732

**Published:** 2022-12-05

**Authors:** Mohammadreza Mohaghegh Neyshabouri, Jens Lagergren

**Affiliations:** 1 Department of Electrical Engineering and Computer Science, KTH Royal Institute of Technology, Stockholm, Sweden; 2 Science for Life Laboratory, Stockholm, Sweden; National Center for Biotechnology Information (NCBI), UNITED STATES

## Abstract

Identifying the interrelations among cancer driver genes and the patterns in which the driver genes get mutated is critical for understanding cancer. In this paper, we study cross-sectional data from cohorts of tumors to identify the cancer-type (or subtype) specific process in which the cancer driver genes accumulate critical mutations. We model this mutation accumulation process using a tree, where each node includes a driver gene or a set of driver genes. A mutation in each node enables its children to have a chance of mutating. This model simultaneously explains the mutual exclusivity patterns observed in mutations in specific cancer genes (by its nodes) and the temporal order of events (by its edges). We introduce a computationally efficient dynamic programming procedure for calculating the likelihood of our noisy datasets and use it to build our Markov Chain Monte Carlo (MCMC) inference algorithm, ToMExO. Together with a set of engineered MCMC moves, our fast likelihood calculations enable us to work with datasets with hundreds of genes and thousands of tumors, which cannot be dealt with using available cancer progression analysis methods. We demonstrate our method’s performance on several synthetic datasets covering various scenarios for cancer progression dynamics. Then, a comparison against two state-of-the-art methods on a moderate-size biological dataset shows the merits of our algorithm in identifying significant and valid patterns. Finally, we present our analyses of several large biological datasets, including colorectal cancer, glioblastoma, and pancreatic cancer. In all the analyses, we validate the results using a set of method-independent metrics testing the causality and significance of the relations identified by ToMExO or competing methods.

## Author summary

Cancer progression is an evolutionary process where somatic mutations in so-called driver genes provide the harboring cells with certain selective advantages. Identifying the interplay among the driver genes is critical for understanding how cancer evolves. In this paper, we introduce a method for analyzing cohorts of tumors. Our approach is based on a novel probabilistic model, which can identify the temporal order of mutations in driver genes, and how they may exhaust each other’s selective advantages. We introduce an efficient likelihood calculation procedure and build an MCMC algorithm for making inferences based on our model. Our computationally efficient inference algorithm enables us to work with hundreds of genes and thousands of tumors. Using a broad set of synthetic data experiments, we demonstrate the performance of our inference algorithm in various scenarios. We also present our analyses of several biological datasets. Our results agree with a set of well-known relations among the driver genes and suggest new interesting such relationships.

This is a *PLOS Computational Biology* Methods paper.

## Introduction

Cancer is a disease caused by evolutionary processes involving the accumulation of somatic mutations in the genome [1]. Some mutations in so-called cancer driver genes confer selective advantages to the cells harboring them. Each driver mutation can potentially affect the chances of happening or fixation for the rest of the driver genes by, for example, exhausting or boosting the selective advantage of mutations in specific genes. Identifying the interplay between driver mutations is crucial for a broad set of research and clinical applications, including choosing targets for new drugs, the prognosis of individual tumors, and designing patient-specific treatment plans. The rapidly increasing amount of publicly available genomics data encourages the development of computational methods to use the rich co-occurrence patterns among the driver mutations to gain a deeper understanding of their effects on each other. In this paper, we introduce a probabilistic cancer progression model for analyzing cross-sectional data from large cohorts of tumors and identifying the mutual exclusivity and chronological patterns among cancer driver genes.

The cancer progression models generally focus on inferring the interactions among the driver mutations. Two main types of such interactions, which have been of particular interest, are the progression and mutual exclusivity relations [2]. The progression relation refers to a situation when a mutation, e.g., a loss-of-function mutation in a tumor suppressor gene, increases the chance of occurrence or fixation of another mutation. The studies on the progression relations among driver event have used various types of structural models including oncogenetic trees [3–9] and conjunctive Bayesian networks (CBN) [10–13]. On the other hand, the mutual exclusivity relation refers to when two mutations are observed together less frequently than expected by chance. This can happen due to the roles of the genes in a particular pathway or protein complex, where a mutation in one of the genes disrupts the functionality of the pathway or protein complex and exhaust the selective advantage of mutation in the rest of the group [14]. Another underlying reason for the mutual exclusivity patterns is a phenomenon called synthetic lethality [15], where the cells can live with mutations in one of the genes in a particular group, but mutations in two or more of the group members lead to the cell death. The mutual exclusivity relations are also studied using various methods, including integer linear programming (ILP) [16], sampling-based methods involving statistical tests [17], and generative probabilistic models [14, 18]. As a set of mutually exclusive driver genes may have the same effect on another gene through disruption of the same biological pathway, simultaneous inference of sets of mutually exclusive drive genes and the progression patterns among these sets can provide a better understanding of cancer’s progression dynamics [2]. An ILP-based algorithm in [19], called PLPM, simultaneously infers mutually exclusive sets of genes called *driver pathways* and a linear progression structure over these pathways. In [20], a probabilistic counterpart of PLPM is introduced. An efficient likelihood calculation procedure within a Markov chain Monte Carlo algorithm enables the probabilistic PLPM [20] to analyze larger datasets. In [2], a method called pathTiMEx is introduced. In pathTiMEx, the cancer progression dynamics are modeled using a CBN with sets of mutually exclusive genes (driver pathways) in its nodes. An iterative EM-like [21] algorithm is used for training the model. Some other studies, including the network aberration model (NAM) [22] and mutual hazard networks (MHN) [23], have taken a rather different approach. In these papers, the cancer progression dynamics are modeled using a base mutation rate for each driver gene and a set of pairwise relations in the form of multiplicative effects that the genes may impose on each other’s mutation rates. While the NAM model [22] only considers the positive (enabling) effects of mutations on each other, the MHN model handles the negative (inhibiting) effects as well.

With the increasing amount of available data, there is a demand for progression models that can handle hundreds of genes and thousands of tumors. The state-of-the-art methods, including pathTiMEx [2] and MHN [23], cannot take more than around 20 genes in the data as their computational complexity grows exponentially with the number of genes. On the other hand, scalable methods such as probabilistic PLPM [20] that can work with many genes have limited modeling abilities. This paper introduces a method called ToMExO (Tree of Mutually Exclusive Oncogenes), which is more scalable than probabilistic PLPM while enjoying a significantly richer modeling ability. Our method ToMExO is still limited compared to pathTiMEx and MHN in the sense of the modeling power. However, as shown in our comparisons in the paper, the progression model found by our algorithm can be better validated even against pathTiMEx and MHN on a dataset feasible to be analyzed by them.

ToMExO is a probabilistic cancer progression method working with datasets including information on the presence/absence of mutations in a set of genes in a cohort of tumors. We simultaneously identify critical driver genes, group them as sets of mutually exclusive genes (driver pathways), and arrange them in a tree structure representing the order in which they get mutated. We introduce a computationally efficient dynamic programming procedure for likelihood calculations, used as the core of a Markov Chain Monte Carlo (MCMC) algorithm to make inferences based on our model. We have also designed a set of novel structural moves, enabling us to explore our model space efficiently. This work improves the probabilistic PLPM method [20] in several ways. Firstly, the modeling power is improved from a single linear chain to a tree-like structure. Furthermore, while [20] uses a grid search strategy for selecting the model complexity (length of the linear structure in their model), ToMExO resolves the model selection issues by introducing flexible MCMC moves that split and merge nodes in various ways. As a result, ToMExO samples can get more or less complex on the fly. Similar to the model used in [20], our model in this paper allows for false positive and false negative errors in the dataset. Although the parameters for the probability of each of these error types can be sampled in the MCMC iterations (the way [20] does it), we introduce a dynamic programming procedure to find the minimum possible values for these parameters, given a fixed structure. We use this procedure inside the MCMC iterations to speed up the training algorithm. After finding the proper structure, we fine-tune the parameters using gradient descent. This approach for dealing with the noise in ToMExO leads to an improved computational complexity compared to the probabilistic PLPM.

The paper is organized into two main sections as follows. Section 1 describes the method, where we start with introducing our cancer progression model in section 1.1. We continue with describing the probabilistic model for our data generation process in section 1.2 and introducing our likelihood calculation algorithm in section 1.3. We finish the method section by explaining our inference algorithm in section 1.4. Section 2 presents our results on a set of synthetic and biological data experiments. In section 2.1 we demonstrate our performance in a set of synthetic data simulations. We present our biological data analyses in section 2.2. In this section, after comparing ToMExO against two state-of-the-art methods on a moderate-size dataset, we extend our experiments to a set of larger biological datasets that the competitor algorithms couldn’t analyze due to the larger number of genes.

## 1 Method

In this section, we start with introducing our model for cancer progression using an example in section 1.1. In section 1.2 we explain how such a progression model, together with a noise injection procedure can form a *generative process* for our cancer dataset. Our objective is to *learn* a progression model from the datasets. To this end, we start with introducing a likelihood calculation algorithm in section 1.3. This algorithm can be seen as a way to evaluate how well each progression model explains the dataset. In section 1.4 we introduce our inference algorithm, which uses our efficient likelihood calculation procedure as the heart of an MCMC sampling scheme to generate samples from the posterior distribution and *learn* the maximum a posteriori (MAP) estimate of the progression model for the input dataset.

### 1.1 Cancer progression model

**Fig 1:**
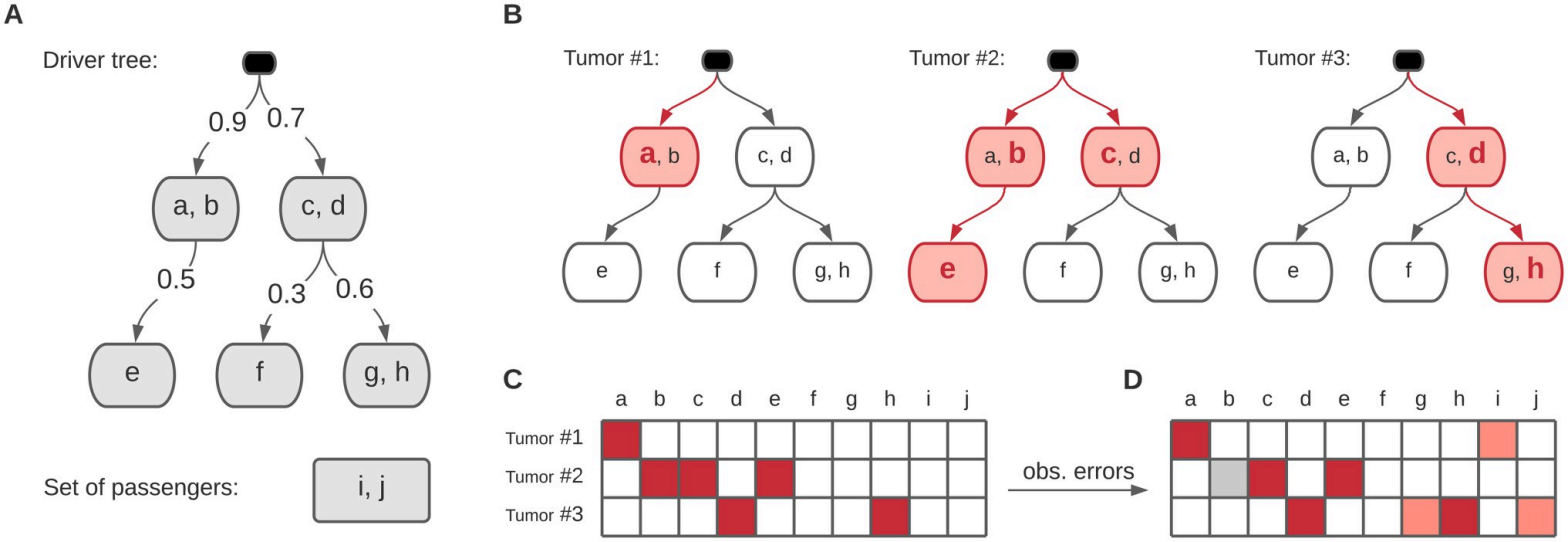
**A**. An example cancer progression model. **B**. Three tumors evolved following the model. The *firing* edges are shown in red. The mutated nodes are filled in red, and the genes with driver mutations are shown in bold red font. **C**. The binary matrix representation of the driver mutations in the example tumors. The blocks filled in red represent the 1’s. **D**. The observed dataset including errors in the forms of false positives (light red) and false negatives (gray).

We model cancer progression using a driver tree (*V*, *E*). Except for the root, the nodes include non-overlapping non-empty subsets of the driver genes. We denote the set of genes in node *v* ∈ *V* by *D*_*v*_. Each edge (*u*, *v*) ∈ *E* has a *firing* probability denoted by *f*_*v*_ ∈ (0, 1). Fig 1A shows an example of a progression model.

The model explains the tumor progressions as follows. Starting from the root, each node has a chance to get *mutated* according to its firing probability. All the mutated nodes get a driver mutation in one and only one of their genes, chosen according to a categorical distribution with parameters proportional to the overall mutation rates of the genes. The children of the mutated nodes may get mutated according to their firing probabilities, and the tumor progresses this way. For example, consider the model shown in Fig 1A. Three tumors that evolved according to this model are shown in Fig 1B. In these example tumors, the mutated nodes are colored in red, and the mutated driver genes are shown using the bold red font.

Given a list of mutations identified in a set of tumors, we can represent the data using a binary matrix with the tumors in the rows and the genes in the columns. Each element (*m*, *n*) is set to one if tumor *m* has a mutation in gene *n*. Following our example scenario in Fig 1, the tumors shown in Fig 1B would result in the matrix shown in Fig 1C, in the absence of any false positives and false negatives. However, the observed data is generally very noisy. The data typically includes many background mutations, which can even happen in the driver genes. Note that when the selective advantage of a mutation in a driver gene is already exhausted by earlier events, we count it as a background mutation. The background mutations and technical errors lead to *false positives* in our data. On the other hand, some driver mutations may be lost due to technical issues such as low coverage of the reads covering those mutations, resulting in *false negatives* in our data. Adding the false positives and false negatives in our example scenario leads to the observed matrix shown in Fig 1D.

### 1.2 Generative process

Let *B* be our binary matrix of shape *M* × *N*, including information on *N* *potentially* driver genes in *M* tumors. The element in the *m*^th^ row and the *n*^th^ column of the matrix, denoted by *B*_*m*,*n*_, is equal to one if and only if the *m*^th^ tumor has at least one mutation in the *n*^th^ gene. We denote the *m*^th^ row and the *n*^th^ column of the matrix by *B*_*m*,:_ and *B*_:,*n*_, respectively.

We call the genes not placed into the driver tree as passenger genes and denote the set of passenger genes by *P*. Each progression model is composed of a driver tree (*V*, *E*), the set of genes in each node {*D*_*v*_}_*v*∈*V*_, the firing probabilities {*f*_*v*_}_*v*∈*V*_, and the set of passengers *P*. We use the notation *T* to refer to the complete set of all variables above, i.e.,

$$\begin{array}{c} T≜(V,E,{\left\{f_{v}\right\}}_{v\in V},{\left\{D_{v}\right\}}_{v\in V},P) \end{array}$$

**Fig 2:**
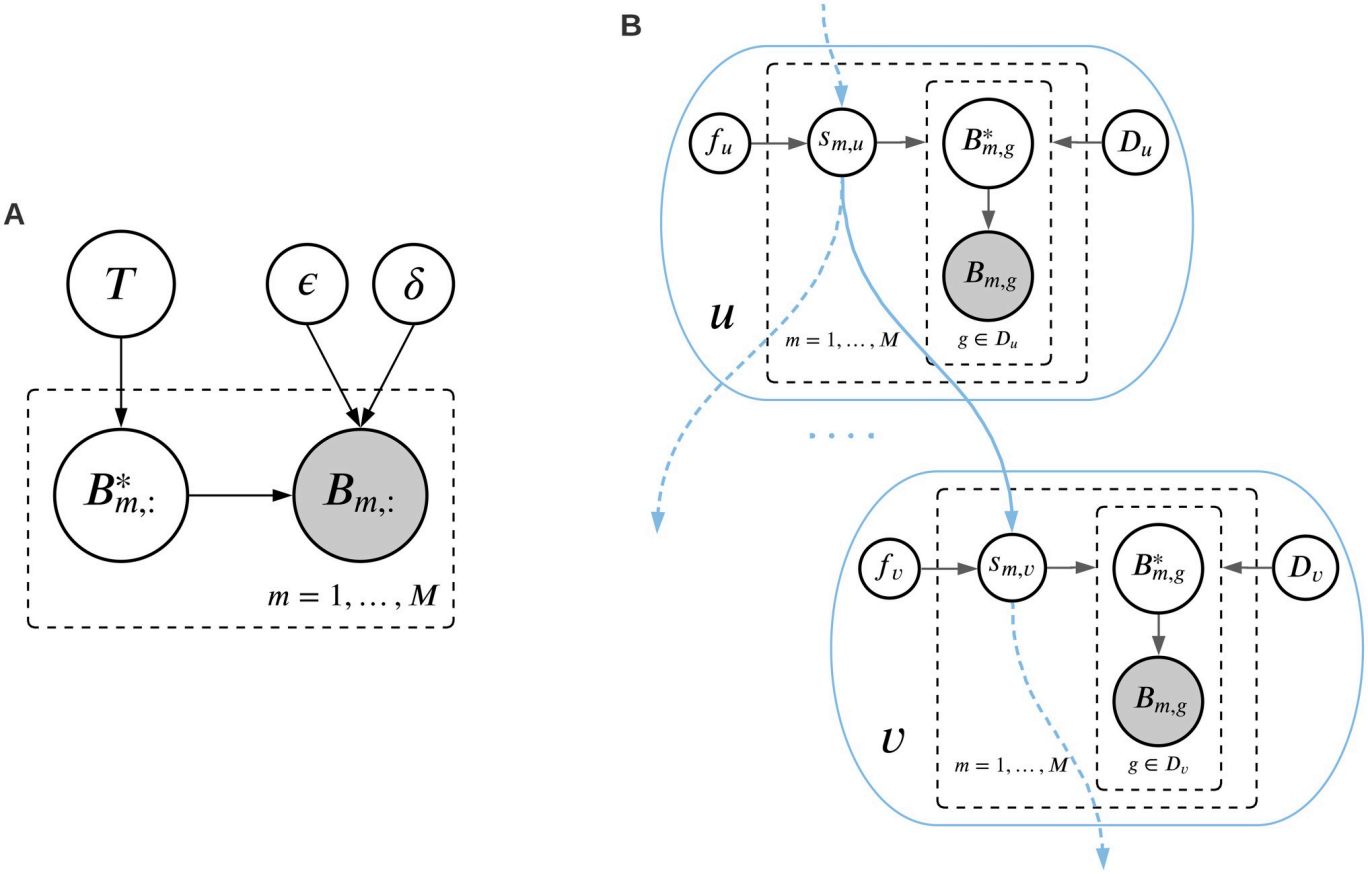
**A**. Graphical model of the generative process, where *T* is the progression model and *ϵ* and *δ* are the probabilities of false positive and false negative, respectively. The latent and observed mutation matrices are denoted by B* and *B*, respectively. **B**. Graphical model of the variables in the nodes of the driver tree. The binary variable *s*_*m*,*u*_ is equal to one if and only if node *u* is mutated in tumor *m*.

A progression model *T*, with false positive probability *ϵ* and false negative probability *δ* form a probabilistic generative model with a graphical model shown in Fig 2A. The matrix B* is the unobserved noise-free dataset. Adding false positives and false negatives to B* results in the observed matrix *B*. To assess how well the dataset conforms with the progression model *T*, *ϵ*, and *δ*, we need to calculate the likelihood *p*(*B*|*T*, *ϵ*, *δ*). The dataset likelihood can be written as the product of the likelihood of the individual tumors, i.e.,

$$\begin{array}{c} p\left(B|T,\epsilon ,\delta \right)=\prod_{m\in \{1,\ldots ,M\}}p\left(B_{m,:}|T,\epsilon ,\delta \right). \end{array}$$ (1)

In order to calculate *p*(*B*_*m*,:_|*T*, *ϵ*, *δ*), we have to marginalize out the unobserved $B_{m,:}^{*}$ vector. We have

$$\begin{array}{c} p\left(B_{m,:}|T,\epsilon ,\delta \right)=\sum_{B_{m,:}^{*}}\;p\left(B_{m,:},B_{m,:}^{*}|T,\epsilon ,\delta \right)=\sum_{B_{m,:}^{*}}\;p\left(B_{m,:}^{*}|T\right)p\left(B_{m,:}|B_{m,:}^{*},\epsilon ,\delta \right). \end{array}$$ (2)

While the summation is to be taken over all 2^*N*^ states of $B_{m,:}^{*}$, not all of these states have non-zero $p(B_{m,:}^{*}|T)$. In the following section, we use this property to introduce an efficient likelihood calculation procedure.

### 1.3 Dynamic programming for likelihood calculations

We use a dynamic programming algorithm over the tree to calculate the dataset likelihood in a computationally efficient manner. To this end, we introduce an auxiliary variables *s*_*m*,*v*_ ∈ {0, 1} in each node *v* to represent the state of *v* in tumor *m* (healthy/mutated). These variables are the ones forming the connection between two nodes *u* and *v* along an edge (*u*, *v*), where we have

$$\begin{array}{c} p\left(s_{m,v}=1|s_{m,u}\right)=\{\begin{array}{cc} f_{v} & ,s_{m,u}=1\\ 0 & ,s_{m,u}=0 \end{array}. \end{array}$$ (3)

The graphical model in Fig 2B shows the dependencies between the variables inside the nodes of a driver tree.

Let $B_{m,D_{v}}$ denote the bits corresponding to the genes in *v*. Similarly, we use $B_{m,D_{v↓}}$ to show the bits corresponding to the genes in *v* and its descendant nodes. In each node *v*, we can calculate the likelihood of $B_{m,D_{v}}$ given both possible states of *s*_*m*,*v*_ as

$$\begin{array}{cc} \Lambda_{m,v} & ≜p\left(B_{m,D_{v}}|s_{m,v}=1\right)=\sum_{B_{m,D_{v}}^{*}}\;p\left(B_{m,D_{v}}^{*}|s_{m,v}=1\right)p\left(B_{m,D_{v}}|B_{m,D_{v}}^{*}\right)\\ & =\frac{\sum_{g\in D_{v}:B_{m,g}=1}\alpha_{g}}{\sum_{g\in D_{v}}\alpha_{g}}\left(1-\delta \right)\epsilon^{o_{m,v}-1}{\left(1-\epsilon \right)}^{z_{m,v}}\\ & +\frac{\sum_{g\in D_{v}:B_{m,g}=0}\alpha_{g}}{\sum_{g\in D_{v}}\alpha_{g}}\delta \epsilon^{o_{m,v}}{\left(1-\epsilon \right)}^{z_{m,v}-1}, \end{array}$$ (4)

$$\begin{array}{cc} \Gamma_{m,v} & ≜p\left(B_{m,D_{v}}|s_{m,v}=0\right)=\sum_{B_{m,D_{v}}^{*}}\;p\left(B_{m,D_{v}}^{*}|s_{m,v}=0\right)p\left(B_{m,D_{v}}|B_{m,D_{v}}^{*}\right)\\ & =\epsilon^{o_{m,v}}{\left(1-\epsilon \right)}^{z_{m,v}}, \end{array}$$ (5)

where *o*_*m*,*v*_ and *z*_*m*,*v*_ are the number of ones and zeros observed in $B_{m,D_{v}}$, and *α*_*g*_ is the mutation rate of *g*. The key part of our algorithm is to calculate the likelihood of $B_{m,D_{v↓}}$ given the state *s*_*m*,*v*_. Let C(v) denote the children set of node *v*. Following a post-order traversal of the tree, we can calculate the likelihoods of type $B_{m,D_{v↓}}$ in each node *v* based on their values in C(v). We have:

$$\begin{array}{cc} \Psi_{m,v} & ≜p\left(B_{m,D_{v↓}}|s_{m,v}=1\right)=p\left(B_{m,D_{v}}|s_{m,v}=1\right)\prod_{c\in C(v)}p\left(B_{m,D_{c↓}}|s_{m,v}=1\right)\\ & =\Lambda_{m,v}\prod_{c\in C(v)}(f_{c}\Psi_{m,c}+\left(1-f_{c}\right)\Omega_{m,c}), \end{array}$$ (6)

$$\begin{array}{cc} \Omega_{m,v} & ≜p\left(B_{m,D_{v↓}}|s_{m,v}=0\right)=p\left(B_{m,D_{v}}|s_{m,v}=0\right)\prod_{c\in C(v)}p\left(B_{m,D_{c↓}}|s_{m,v}=0\right)\\ & =\Gamma_{m,v}\prod_{c\in C(v)}\Omega_{m,c}. \end{array}$$ (7)

Finally, denoting the numbers of ones and zeros observed in *B*_*m*,*P*_ by *o*_*m*,*P*_ and *z*_*m*,*P*_, the likelihood of the *m*^th^ tumor will be:

$$\begin{array}{c} p\left(B_{m,:}|T,\delta ,\epsilon \right)=\Psi_{m,\text{root}}\epsilon^{o_{m,P}}{\left(1-\epsilon \right)}^{z_{m,P}}, \end{array}$$ (8)

which concludes our dynamic programming procedure.

We emphasize that only a single post-order traversal of the driver tree is sufficient for calculating the likelihood, as described in this section. As a result, the computational complexity of our likelihood calculation procedure is linear in the number of driver tree nodes. As the number of nodes cannot exceed the number of genes *N*, our computational complexity is linear in the number of genes in the worst-case scenario. As we calculate the likelihood of our *M* tumors separately, we have a likelihood calculation algorithm with a computational complexity of O(MN).

### 1.4 Inference algorithm

In this section, we introduce our Markov Chain Monte Carlo algorithm for making inferences using our model. Given a dataset *B*, the objective is to find a maximum a posteriori (MAP) estimate of the model *T* and error parameters *ϵ* and *δ*. In this paper, we restrict ourselves to models *T* = (*V*, *E*, {*f*_*v*_}_*v*∈*V*_, {*D*_*v*_}_*v*∈*V*_, *P*) with firing probabilities {*f*_*v*_}_*v*∈*V*_ matching the *empirical estimations*, as explained in the following. Let the topology (*V*, *E*) and the gene assignments ({*D*_*v*_}_*v*∈*V*_ and *P*) be given. To estimate the firing probability of an edge (*u*, *v*), denoted by *f*_*v*_, we denote the number of tumors with mutations in *u* by $X_{B,u}$. Similarly, we denote the number of tumors with mutations in both *v* and its parent *u* by $Y_{B,v}$. We set

$$\begin{array}{c} f_{v}=\;\text{max}\;\{\frac{Y_{B,v}-\epsilon X_{B,u}}{\left(1-\epsilon -\delta \right)X_{B,u}},0\}, \end{array}$$ (9)

which corresponds to a maximum likelihood estimate of the firing probability value. Note that in case of small *ϵ* and *δ*, we have $f_{v}\approx Y_{B,v}/X_{B,u}$. The derivations for this formula can be found in Section 1.4 in S1 Text.

To have a computationally efficient inference algorithm, we further restrict ourselves to error parameters *ϵ* and *δ* tuned to the model *T*. To this end, we use a dynamic programming procedure to calculate the minimum number of false positive and false negative events in the data, given a fixed progression structure. In this way, we can calculate an *empirical estimation* of the parameters *ϵ* and *δ* with a single post-order traversal of the driver tree. Our empirical error estimation algorithm is described in detail in Section 1.1 in S1 Text.

Following a Bayesian inference framework, we use a prior of the form

$$\begin{array}{c} p\left(T\right)={\left(\frac{1}{|S_{B^{*}}\left(T\right)|}\right)}^{\zeta }, \end{array}$$ (10)

where $S_{B^{*}}\left(T\right)$ is the set of possible *noise-free* tumor vectors, i.e., $B_{m,:}^{*}$, which can be constructed using *T*, and $|S_{B^{*}}\left(T\right)|$ is the cardinality of this set. The parameter *ζ* can adjust our penalty for higher $|S_{B^{*}}\left(T\right)|$ values. We have used *ζ* = 5 as a default parameter in all the synthetic and biological data analyses presented in the paper.

**Fig 3:**
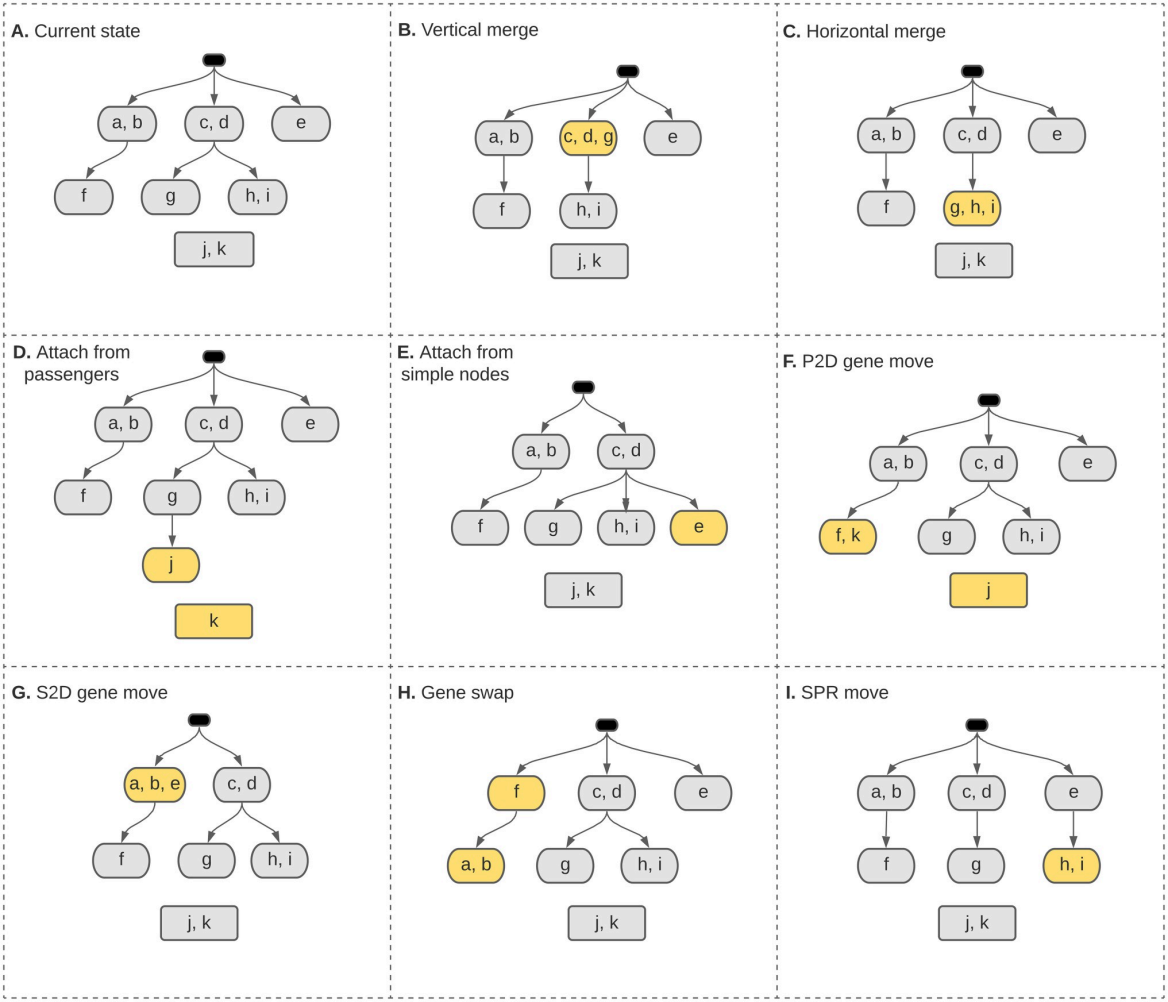
Different types of structural moves used in our MCMC inference algorithm.

We initialize our MCMC sampler to a star tree, where each gene has its own node in the first layer of the driver tree. We call such single-gene nodes in the first layer of the driver tree as *simple nodes*. Following a Metropolis-Hasting framework, we use a set of possible structural moves to propose new candidate trees. The proposed trees may then get accepted based on their Metropolis-Hasting acceptance ratio. To explore the space of progression models in an efficient way, we have designed several types of structural moves, including various topological moves, as well as gene assignment modifications. Fig 3 shows a few example moves. Our structural moves include:

- “Vertical merge”, merging a leaf node into its parent (Fig 3B), and its reverse move called “vertical split”,
- “Horizontal merge”, merging two sibling leaves (Fig 3C), and its reverse move called “horizontal split”,
- “Attach from passengers”, attaching a new node containing a subset of the passenger genes to the driver tree (Fig 3D), and its reverse move called “detach into passengers”,
- “Attach from simple nodes”, attaching a new node containing the genes from a subset of the simple nodes to the driver tree (Fig 3E), and its reverse move called “detach into simple nodes”,
- “P2D gene move”, moving a single gene from the set of passengers to an existing node in the driver tree (Fig 3F), and its reverse move called “D2P gene move”,
- “S2D gene move”, moving the gene in a simple node into an existing node of the driver tree (Fig 3G), and its reverse move called “D2S gene move”,
- “Gene swap”, swapping the genes between a node and its parent (Fig 3H),
- “SPR”, subtree pruning and regrafting to modify the driver tree structure (Fig 3I).

After generating a set of samples, we select the sample with the maximum posterior. We fine-tune the error parameters *ϵ* and *δ* using gradient descent with a few iterations over the selected driver tree. A pseudo-code of the inference algorithm, together with detailed explanations of the components of our algorithm, are included in Section 1 in S1 Text. We emphasize that as the individual components within each of our MCMC iterations have linear computational complexities in the size of the dataset, we have an inference algorithm with linear per-iteration computational complexity.

## 2 Results

### 2.1 Synthetic data experiments

**Fig 4:**
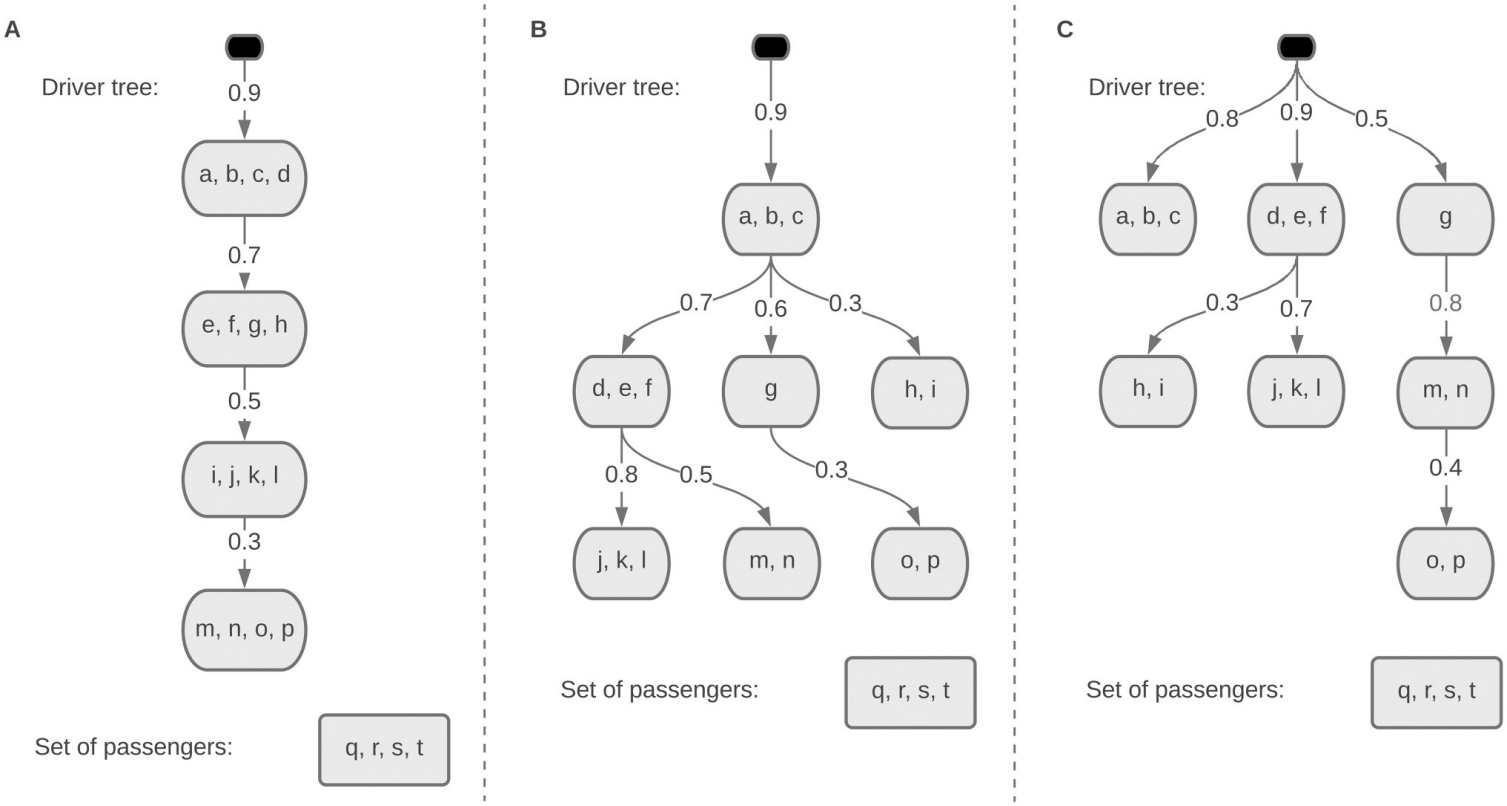
Generative progression model for the synthetic data experiments with **A**. linear model **B**. single-seeded tree model **C**. multi-seeded tree model.

In this section, we use synthetic data simulations to demonstrate the efficiency of our inference algorithm in three example scenarios with generative progression models shown in Fig 4. The linear model shown in Fig 4A is an example of models called *Pathway Linear Progression Models* studied in [19] and [20], where the progression follows a linear evolutionary path over so-called driver pathways. The single-seeded tree model shown in Fig 4B represents the scenarios where the cancer is always initiated from a single critical driver pathway but can progress along different trajectories in a tree-like structure. The multi-seeded tree model shown in Fig 4C represents a further generalization, where different driver pathways can independently initiate and drive the cancer progression.

For each generative progression model, we explored 16 settings, with equal false positive and false negative probabilities in {0.001, 0.01, 0.05, 0.1} and the number of tumors in {50, 100, 200, 500}. We sampled 10 datasets for each setting using the generative process explained in section 1.2. We have used our inference algorithm with 100*k* iterations throughout our synthetic data experiments.

#### Evaluation metrics for synthetic data analysis

We can compare our results against the known generative progression models to evaluate our performance during the synthetic data experiments. To this end, we focus on the sets of mutual exclusivity and progression relations implied by the models. If two genes *g*_1_ and *g*_2_ are placed together in a common node of a model *T*, we say the pair (*g*_1_, *g*_2_) is in the set of mutual exclusivity relations implied by *T*. Similarly, if the node including *g*_1_ is an ancestor of the node including *g*_2_, we say (*g*_1_, *g*_2_) is in the set of progression relations implied by *T*. We calculate our precision and recall in recovering these two sets of relations and calculate two F-scores. Let *F*_ME_ be our F-score for identifying the mutual exclusivity relations. Similarly, let *F*_PR_ be our F-score for identifying the progression relations. We define our overall score, denoted by *F*_overall_, as the harmonic mean of *F*_ME_ and *F*_PR_, i.e.,

$$\begin{array}{c} F_{\text{overall}}=\frac{2*F_{\text{ME}}*F_{\text{PR}}}{F_{\text{ME}}+F_{\text{PR}}}. \end{array}$$ (11)

Note that *F*_overall_ ∈ [0, 1] and *F*_overall_ = 1 implies perfect identification of the generative model, while the star tree (which is our initial state) has *F*_overall_ = 0, as its recall is zero for both mutual exclusivity and progression relations.

#### Performance on synthetic data

**Fig 5:**
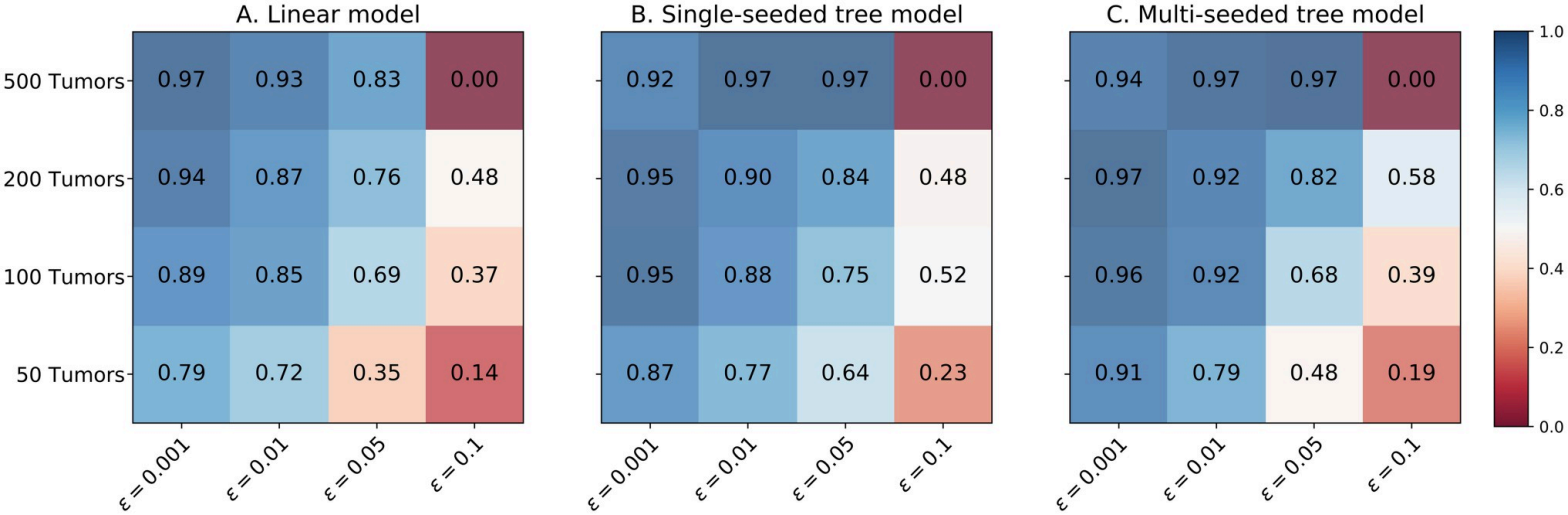
The averaged *F*_overall_ scores achieved in the synthetic data experiments in the case of **A**. linear model **B**. single-seeded tree model **C**. multi-seeded tree model.

Fig 5 shows the F-scores achieved by our inference algorithm (averaged over our 10 datasets for each case). As shown in this figure, our inference algorithm finds the exact generative model, or a very similar one, for all the cases with error probabilities up to 0.05 and at least 100 tumors in the dataset. The figure shows that the performance improves with increments in the number of tumors and reductions in noise. These analyses also show our method’s limitations in dealing with datasets having an error probability of 0.1. We have provided an extensive supplementary analysis of the synthetic experiments in Section 2 in S1 Text. Our analyses suggest two plausible explanations for our poor *F*_overall_ scores in the cases with *ϵ* = *δ* = 0.1. Firstly, when the error rate is this high, the star tree (our MCMC initial state) provides a competitive posterior against the generative model. As a result, it’s much harder to improve the posterior with small modifications, leading to the chains getting stuck close to the initial state. We also observe that the *F*_overall_ score is a very fragile metric that can easily drop with the slightest errors in the recovered progression model.

Further details on our evaluation metrics, the individual precision and recall values, the performance in terms of likelihoods, and the inferred error values are provided in Section 2 in S1 Text. We have also included our performance measured by two distance metrics designed for phylogenetic trees (DISC and CASET), introduced in [24]. This section in the supplementary document also includes information on the run-times of our synthetic data experiments, reaffirming that our computational complexity barely depends on the noise level or the driver tree structure and increases linearly with the size of the dataset.

### 2.2 Biological data analysis

In this section, we present our experiments on several biological datasets. We start this section by explaining our metrics to assess the results. We then compare our method ToMExO against two state-of-the-art methods, pathTiMEx [2] and MHN [23] on a moderate-size glioblastoma dataset. Finally, we present our analysis on larger biological datasets, which are not practical to study using pathTiMEx or MHN.

#### Evaluation metrics for biological data analysis

To have a method-independent metric to assess the progression models, we use probabilistic causation tests introduced in [8] as explained in the following:

- **Progression scores**: if node *u* is a parent of node *v*, mutation in *u* should increase the chance of mutation in *v*. We define our progression score as
  $$\begin{array}{c} \lambda_{\text{PR}}\left(u,v\right)=\frac{p\left(v|u\right)-p\left(v|\bar{u}\right)}{p\left(v|u\right)+p\left(v|\bar{u}\right)}, \end{array}$$ (12)
  where *p*(*v*|*u*) and $p(v|\bar{u})$ are the rates of observing a mutation in *v*, among the tumors with and without mutation in *u*, respectively. Note that if a mutation in *u* increases the chance of mutation in *v*, then a mutation in *v* also increases the chance of mutation in *u*. The progression score defined in Eq (12) can be used to find the causation direction for such a relationship between *u* and *v* [8]. We have λ_PR_(*u*, *v*) ∈ [−1, 1] and in the case of λ_PR_(*u*, *v*) > 0, a mutation in *u* or *v* increase the chance of mutation in the other one. As discussed in [8], the amplitude of λ_PR_ shows the strength of the relationship and when λ_PR_(*u*, *v*) > λ_PR_(*v*, *u*), it’s more plausible to say *u* is the cause of *v* than the other way around.
- **Mutual exclusivity scores**: Following the same idea as the progression score, if two genes *g*_1_ and *g*_2_ are mutually exclusive, they should reduce each other’s chance of mutation. We define the mutual exclusivity score of a pair of genes (*g*, *w*) as the average strength of the mutual exclusivity signal in both directions:
  $$\begin{array}{c} \lambda_{\text{ME}}\left(g,w\right)=0.5*(\frac{p\left(g|\bar{w}\right)-p\left(g|w\right)}{p\left(g|\bar{w}\right)+p\left(g|w\right)}+\frac{p\left(w|\bar{g}\right)-p\left(w|g\right)}{p\left(w|\bar{g}\right)+p\left(w|g\right)}). \end{array}$$ (13)
  We define the score of a node *u* (including mutually exclusive genes) as the average λ_ME_ among the pairs of genes in *u* and denote it by λ_ME_(*u*). Note that λ_ME_(*u*) ∈ [−1, 1] and if λ_ME_(*u*) > 0, the genes in *u* have some levels of mutual exclusivity, depending on how high λ_ME_(*u*) is.

In the following, we define p-values for the progression and mutual exclusivity signals to check how likely it is that the observed patterns have happened by chance. Starting from the progression patterns, let (*u*, *v*) be an edge with positive λ_PR_, where λ_PR_(*u*, *v*) > λ_PR_(*v*, *u*). Consider a null hypothesis that says *v* is independent of *u* and gets mutated with a probability equal to *p*(*v*) in each tumor (*p*(*v*) is the empirical mutation rate of *v* across all tumors). Having observed *n*_*u*,*v*_ tumors with mutations in both *u* and *v*, we can calculate how likely it is that the null hypothesis generates *n*_*u*,*v*_ or more tumors with a mutation in *v*, among the tumors which have a mutation in *u*, i.e.,

pPR(u,v)≜∑i=nu,vnu(nui)p(v)i(1-p(v))nu-i. (14)

For the mutual exclusivity relation among a pair of genes, we consider the two directions of the relation separately. Assume that a mutation in gene *g* reduces the chance of mutation in gene *w*. Consider a null hypothesis that *w* gets mutated independently with a probability equal to *p*(*w*). Having *n*_*g*,*w*_ tumors with mutations in both *g* and *w*, we can calculate how likely it is that the null hypothesis generates *n*_*g*,*w*_ or fewer tumors with a mutation in *w* among the tumors which have a mutation in *g*:

pME(g,w)≜∑i=0ng,w(ngi)p(w)i(1-p(w))ng-i. (15)

We define the p-value of the mutual exclusivity relation to be the average of the p-values in both directions. We also define the p-value of a mutually exclusive set of genes as the average of the p-values among its pairs of genes.

#### Comparison against pathTiMEx and MHN

We used ToMExO to analyze a glioblastoma dataset previously analyzed by pathTiMEx [2] and MHN [23]. This dataset is originally from The Cancer Genome Atlas (TCGA) [25], pre-processed as explained in [14]. The dataset includes 261 tumors and 20 events, including point mutations, amplifications, and deletions. We downloaded the pre-processed dataset from the MHN public repository [23]. We ran ToMExO with 10 MCMC chains and 100*k* samples and reported the sample with the maximum posterior among all visited samples.

**Fig 6:**
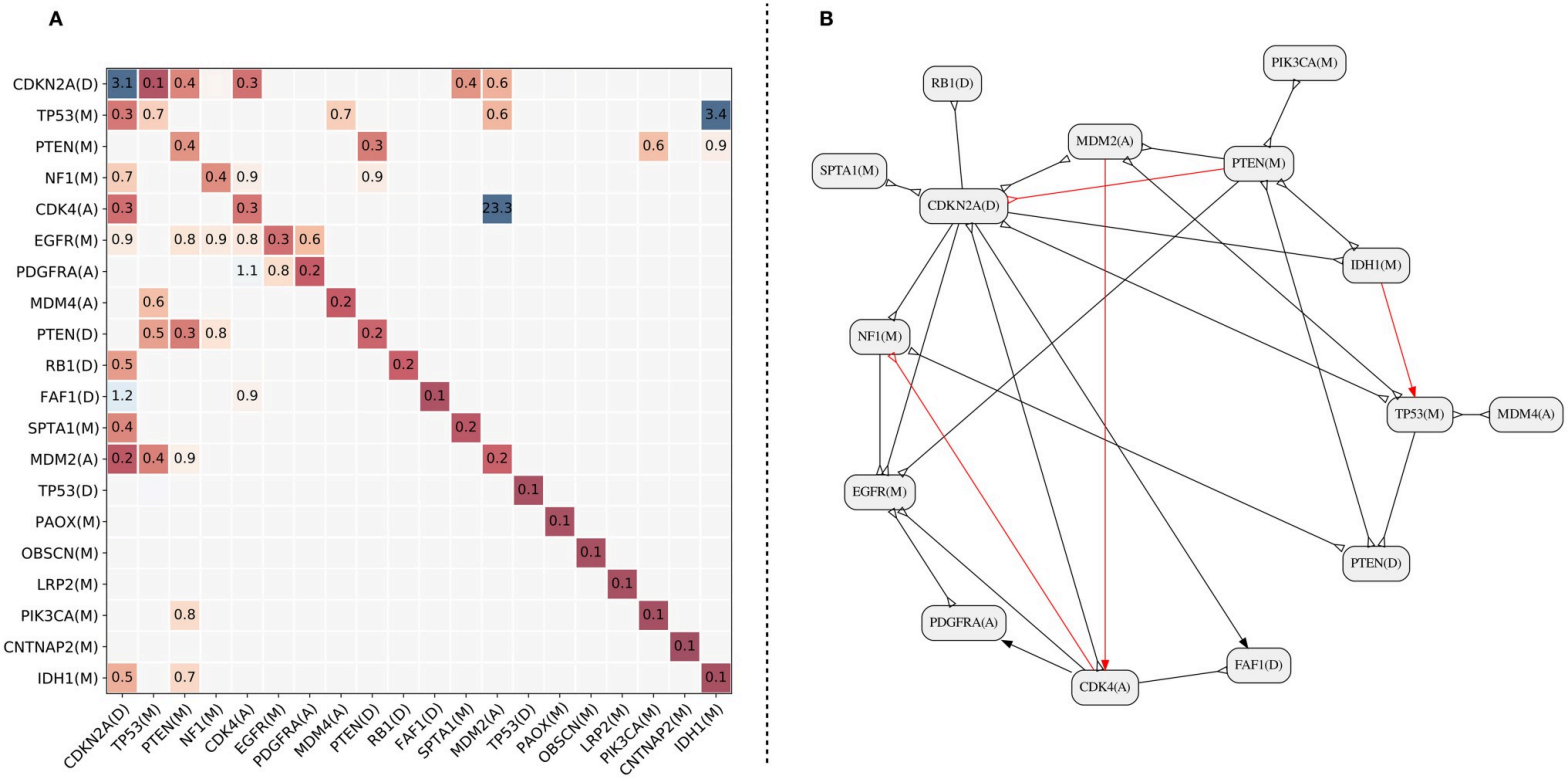
MHN result for the pathTiMEx Glioblastoma dataset. **A**. The matrix of base rates and multiplicative factors. **B**. The corresponding graph. Note that the disconnected nodes are not plotted.

Fig 6A shows the Mutual Hazard Network (MHN) produced by the MHN method. In the MHN model, each event has a base rate of occurrence (shown in the diagonal). Some events have specific multiplicative effects on a set of other events. Such effects can be inhibition, with a factor smaller than one, or promotion, otherwise. In the MHN matrix shown in Fig 6A, the number shown in row *r* and column *c* shows the effect of event *c* on event *r*. The interrelations coded by the MHN can be shown using a graph. Fig 6B shows the graph corresponding to the MHN in Fig 6A. In this graph, the normal arrowheads represent the promoting effects (multiplicative factors higher than one), and the inverted arrowheads represent the inhibiting effects (factors less than one). We evaluated the MHN edges by the progression score discussed above. The one-directional edges that are reported in a wrong direction are colored in red (see Fig 6B). The figure shows that 4 edges (out of 16 one-directional edges) are reported in the reverse direction. We emphasize that the edges colored in red are not invalid. For instance, observing the event MDM2(A) does increase the chance of observing CDK4(A) (see the positive progression score). However, if we were to report a single direction for this relationship, it is more plausible to say CDK4(A) promotes MDM2(A) than the other way around.

**Fig 7:**
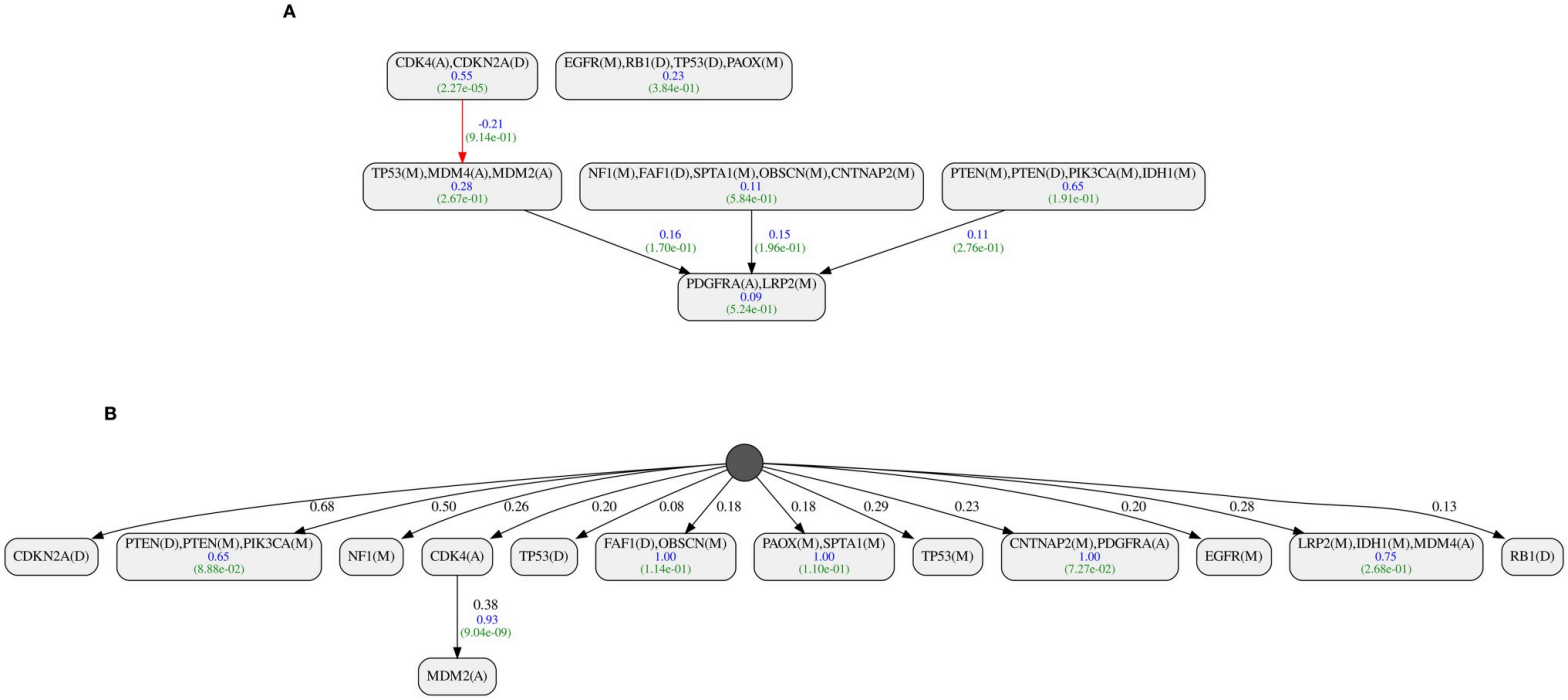
**A**. PathTiMEx result for the pathTiMEx Glioblastoma dataset. **B**. ToMExO result for the pathTiMEx Glioblastoma dataset.

Fig 7 shows the results of analyzing the same dataset using pathTiMEx and ToMExO. The nodes’ mutual exclusivity scores and the edges’ progression scores are shown in blue. The p-value of the mutual exclusivity and progression scores are shown in green below the corresponding scores. Comparing the mutually exclusive sets reported by ToMExO and pathTiMEx, we can see that ToMExO sets have significantly higher mutual exclusivity scores and better p-values on average. As shown in Fig 7A, one of the edges reported by pathTiMEx (colored in red) has a negative progression score. This means that a parent mutation actually decreases the child’s chance of mutation. The other edges reported by pathTiMEx are valid (the parent promotes the child) and in the correct direction. Unlike our model, the model used in pathTiMEx allows for more than one parent for each node. The pathTiMEx model implies that all the parents of a node have to get mutated before the node gets a mutation. As shown in the figure, one of the nodes in the pathTiMEx result has three parents. We checked the progression relation between the parents’ product (logical AND) and this multi-parent node. Interestingly, the resulting progression score is 0.27 from the parents to the child, but 0.32 in the reverse direction, which suggests that the child node is the cause of the parents. We refer to [26] for a comprehensive discussion on these kinds of hypothesis testings on causation relations. Our method, ToMExO, only reports one progression relation, which is very strong (λ_*PR*_ = 0.93), in the correct direction, with a pretty significant p-value. Note that the same relation, but in the opposite direction, was reported by the MHN.

#### Analysis of somatic mutations in TCGA datasets

In the following, we present a selection of our analysis on a set of larger TCGA datasets, which are impossible to investigate by MHN or pathTiMEx due to the large numbers of genes (mutation events) in the data. We downloaded mutation-called TCGA data from the GDAC firehose and limited our focus to the somatic mutations in the cancer-type specific lists of driver genes suggested by IntOGen [27]. Note that the IntOGen pipeline selects the list of driver genes considering a broad set of features, including the rate of mutations, linear and 3D clustering of mutations in individual genes, trinucleotide-specific biases, and functional impacts of individual mutations.

**Fig 8:**
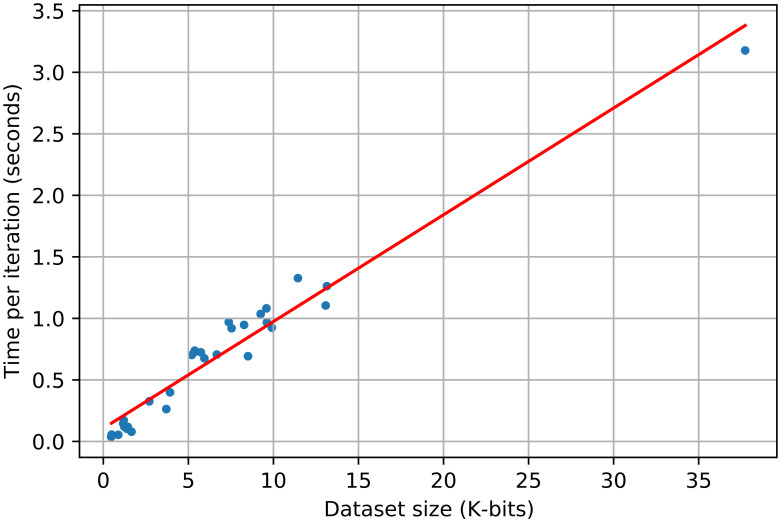
The per-iteration computational complexity of ToMExO with respect to the dataset size (number of tumors × number of events).

To construct our binary input matrix, we consider a tumor mutated in a gene if it has at least one non-silent mutation. Similar to the previous section, we ran ToMExO with 10 MCMC chains and 100*k* samples and reported the maximum a posteriori sample in all the following analyses. We analyzed all the 30 cancer types available in GDAC firehose. Fig 8 shows the computation time for all of our datasets in this part. As shown in this figure, our computational complexity is linear in the size of the dataset, as expected. In the following, we present a selection of our results, while the rest are available in our GitHub repository.

#### Glioblastoma multiforme (GBM)

**Fig 9:**
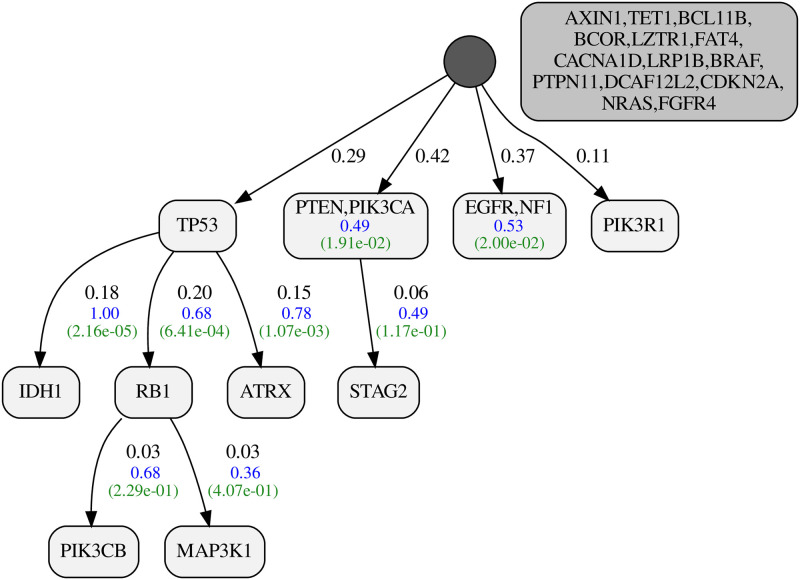
Inferred progression model for Glioblastoma multiforme (GBM). The figure shows the mutual exclusivity score of the nodes (with at least two genes) and the progression score of the edges in blue. Below each score, we show the p-value for the corresponding signal in green. The darker node beside the driver tree represents the set of passenger genes.

Our GBM dataset includes 290 tumors and 26 genes. The resulting progression model is shown in Fig 9. Our inferred values for false positive and false negative probabilities are 0.016 and 0.040, respectively. The posterior ratio of the resulting model to the star tree is 3.49 * 10^37^, corresponding to a per-tumor ratio of 1.347. The GBM dataset with more than 7.5 thousand bits can fully conform with our progression model, assuming only 17 false positives and 77 false negatives.

As shown in Fig 9, our progression model for glioblastoma has PTEN and PIK3CA together in the first layer of the driver tree. These two genes are known to play essential roles in the so-called PTEN–PI3K axis [28] balancing the cell growth. As a mutation in either of these genes can break the balance, it seems plausible that they exhaust each other’s selective advantage, leading to the observed mutual exclusivity pattern between them. Interestingly, the discussions in [28] suggest different treatment approaches for tumors with mutations in PTEN or PIK3CA. Mutations in STAG2 are known to be selected during tumorigenesis [29]. We identified a significant over-representation of STAG2 mutations among the tumors with mutated PTEN. This relation is encoded by the edge from PTEN/PIK3CA to STAG2 in the progression model.

The other multi-gene node in the first layer includes EGFR and NF1, which are considered the main drivers of the classical and mesenchymal subtypes of GBM, respectively [30]. These two genes are expected to show patterns of mutual exclusivity as the classical subtype usually lacks mutations in NF1. The other subtype, proneural, is associated with mutations in TP53 and IDH1 [31]. Mutations in these two genes are also common in the mesenchymal subtype but not in the classical [32]. The progression relation from TP53 to IDH1 is a well-known relation with clinical significance [33]. The timeline of GBM tumor development introduced in [34] suggests mutations in TP53 and EGFR as early clonal events, followed later by IDH1. In the following, we explain how the ToMExO model resolves an important ambiguity in this interpretation. Our progression model for GBM recovers the perfect progression relation (λ_*PR*_ = 1) from TP53 to IDH1. However, EGFR is placed in a separate first-layer node. We tested the progression relation from EGFR to IDH1 and found a significant negative progression score (λ_*PR*_ = −0.67), which means that mutations in EGFR highly reduce the chance of mutation in IDH1. This was expected since mutations in TP53 and IDH1 are not common in the classical subtype, where EGFR is highly mutated, as mentioned above. We emphasize that the other significant progression relation of our model, from TP53 to RB1, is also interesting as these two tumor suppressors are known to cooperate in glioblastoma tumorigenesis [35].

#### Colorectal Adenocarcinoma (COADREAD)

**Fig 10:**
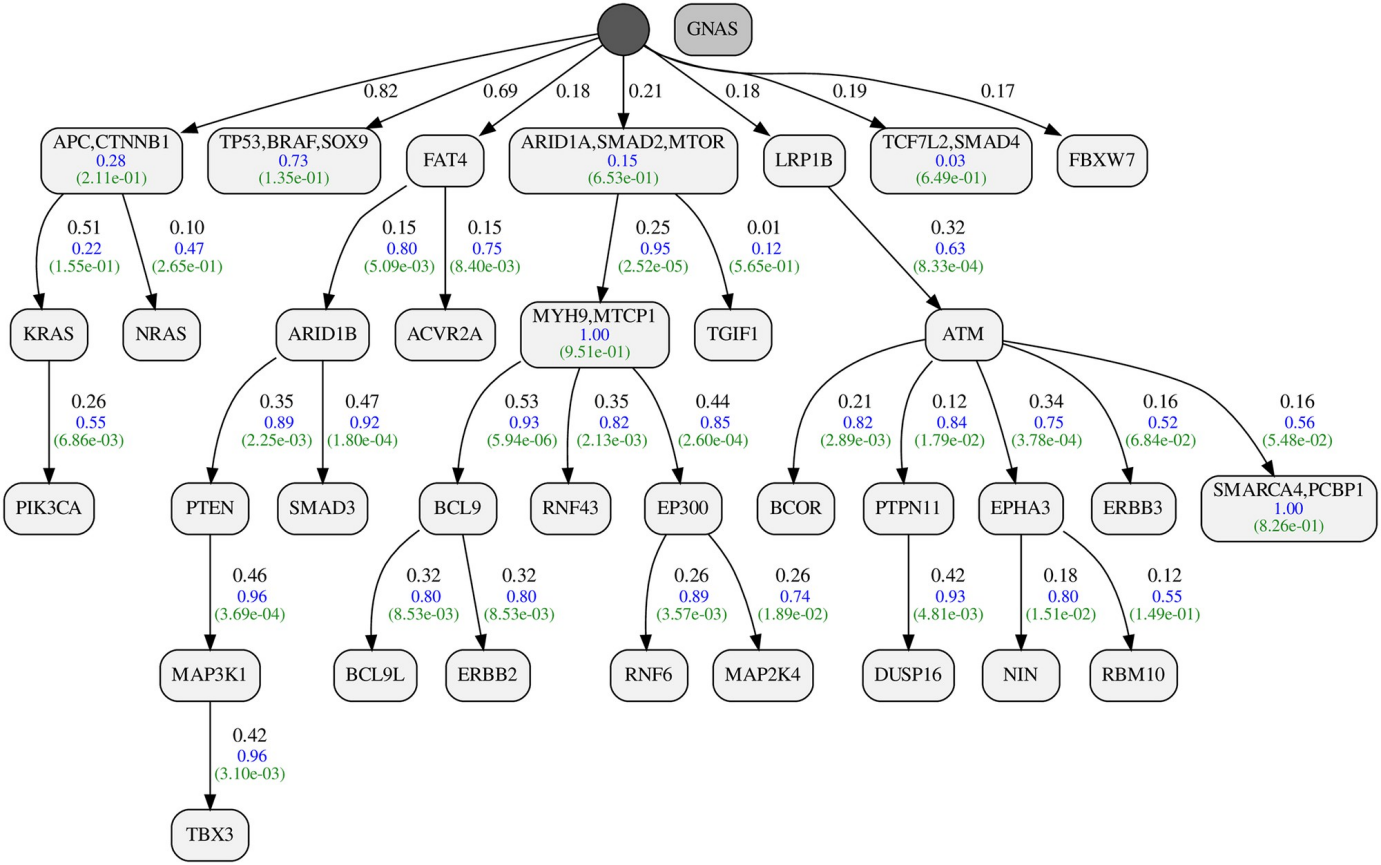
Inferred progression model for Colorectal Adenocarcinoma (COADREAD).

Our COADREAD dataset includes 223 tumors and 43 genes. The resulting progression model is shown in Fig 10. Our inferred false positive and false negative probabilities are 0.022 and 0.076, respectively. The posterior ratio of the resulting model to the star tree is 2.41 * 10^78^, corresponding to a per-tumor ratio of 2.246. The dataset with more than 9.5 thousand bits can fully conform with our progression assuming 67 false positives and 107 false negatives.

As recovered by our progression model, KRAS mutations in colorectal cancer are known to happen after mutations in APC [36]. Mutations in TP53 are also shown to play important oncogenetic roles in colorectal cancers [37]. ToMExO puts TP53 in a node separated from APC → KRAS → PIK3CA chain. The interrelations between TP53, APC and KRAS in colorectal cancer have been of particular interest and investigated using various models starting from the classical Fearon and Vogelstein’s model [38], suggesting APC → KRAS → TP53 progression pattern. We tested the progression scores between TP53 and both APC and KRAS. We see that in our dataset, we have λ_*PR*_(KRAS → TP53) = −0.06 and λ_*PR*_(APC → TP53) = 0.02. Therefore, TP53 is *almost* independent of APC and KRAS, as suggested by our progression model.

Our progression model for colorectal cancer (Fig 10) includes many interesting mutual exclusivity and progression patterns, including the mutual exclusivity of (TP53, BRAF, SOX9) and progression relations from (ARID1A,SMAD2,MTOR) to MYH9 to BCL9, which is a repeated pattern in the data.

#### Pancreatic adenocarcinoma (PAAD)

**Fig 11:**
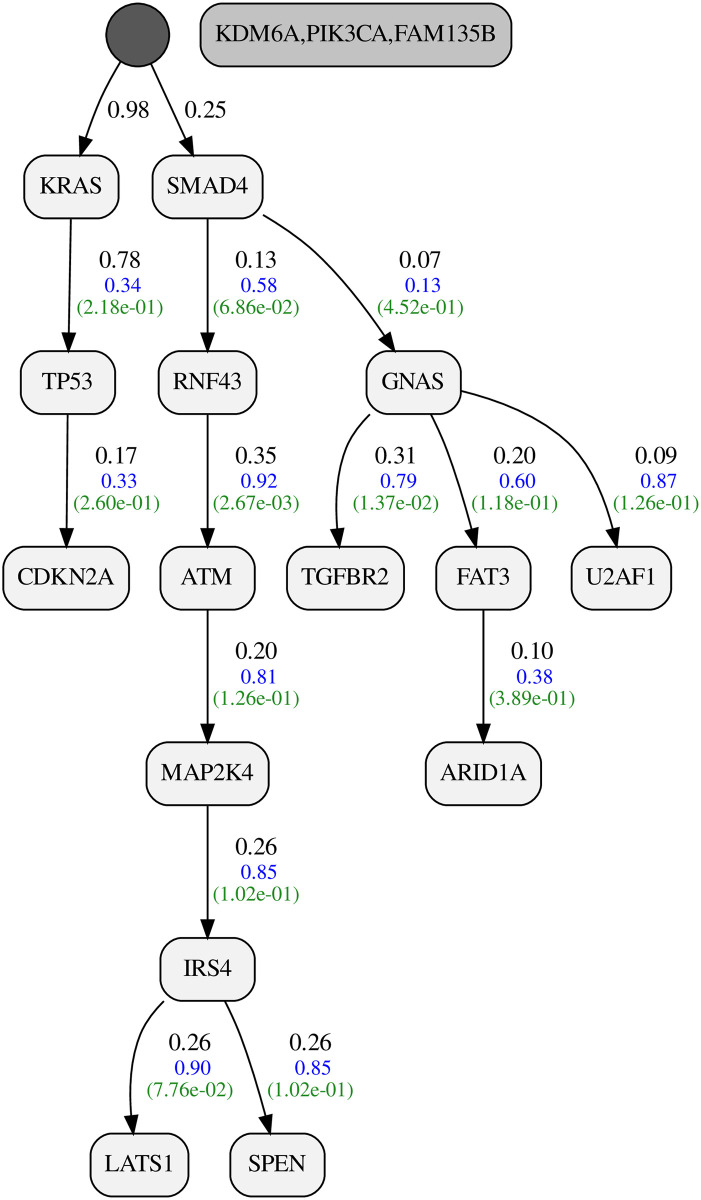
Inferred progression model for Pancreatic adenocarcinoma (PAAD).

Our PAAD dataset includes 150 tumors and 18 genes. The resulting progression model is shown in Fig 11. Our inferred false positive and false negative probabilities are 0.025 and 0.072, respectively. The posterior ratio of the resulting model to the star tree is 1.11 * 10^21^, corresponding to a per-tumor ratio of 1.381. The dataset with 2.7 thousand bits can fully conform with our progression, assuming 26 false positives and 40 false negatives.

Mutations in KRAS and TP53 are present in 91 and 69 percent of our samples, respectively. These two genes are known to be highly associated with oncogenesis in pancreatic cancer [39]. We have CDKN2A later in the chain, which agrees with the tumor development timeline suggested by [34], where KRAS and TP53 are shown to be early clonal events with CDKN2A following later.

In another branch independent from KRAS and TP53, we have SMAD4. We calculated a progression score of 0.08 from KRAS to SMAD4, which agrees with the separated branch for SMAD4. The mutations in SMAD4 are well-known to be important in pancreatic cancer [40]. Our model suggests interesting progression relations from SMAD4 to RNF43 and from NF43 to ATM, which might be interesting for further studies.

## Discussion

In this paper, we introduced ToMExO, a probabilistic method for analyzing cross-sectional mutation data from cohorts of tumors. Our method is designed to simultaneously infer the mutual exclusivity patterns among the driver genes and place them into a tree-like structure coding the temporal order of events. We introduced a computationally efficient inference algorithm for investigating huge datasets with hundreds of genes and thousands of tumors. After an extensive set of synthetic data experiments, we presented our results on a dataset previously analyzed by two state-of-the-art methods, showing our superior performance. Finally, we presented our analyses on larger TCGA datasets that the opponents cannot approach due to the large numbers of genes in the datasets. Our inferred progression models for glioblastoma, colorectal, and pancreatic cancers presented in this paper recover several well-known mutual exclusivity and progression patterns among specific genes and suggest a broad set of new such relations. ToMExO is publicly available in our GitHub repository (https://github.com/mrmohaghegh/tomexo). Our complementary analyses of another set of 27 cancer types can be found in our repository.

While this paper was focused on inferring the progression and mutual exclusivity patterns at the level of tumors, it’s worth mentioning that our method is also applicable at the level of individual clones. The mutually exclusive mutations may happen in different clones of a single tumor. Moreover, various clones of a tumor may follow different evolutionary paths. Therefore, considering the clonal structure of the tumors seems appealing for further investigations in future studies.

## Supporting information

S1 Text Supplementary details. (PDF)
