# Supplementary material for: ToMExO: A probabilistic tree-structured model for cancer progression
Source: PLoS Comput Biol. 2022 Dec 5;18(12):e1010732. doi: 10.1371/journal.pcbi.1010732 (PMC9754607; doi:10.1371/journal.pcbi.1010732)
Supplement: S1 Text — (PDF) [file pcbi.1010732.s001.pdf]

ToMExO: A probabilistic tree-structured model for cancer progression:
Supplementary details

Mohammadreza Mohaghegh Neyshabouri, Jens Lagergren

Contents

1 Method details **2**
1.1 Empirical Error Estimation . . . 2
1.2 Gradient Descent for Error Estimation . . . 2
1.3 Structural Moves . . . 4
1.3.1 Vertical merge . . . 4
1.3.2 Vertical split . . . 4
1.3.3 Horizontal merge . . . 5
1.3.4 Horizontal split . . . 5
1.3.5 Attach from Passengers . . . 5
1.3.6 Detach into Passengers . . . 6
1.3.7 P2D gene move . . . 6
1.3.8 D2P gene move . . . 6
1.3.9 Attach from simple nodes . . . 6
1.3.10 Detach into simple nodes . . . 6
1.3.11 S2D gene move . . . 6
1.3.12 D2S gene move . . . 7
1.3.13 Gene swap . . . 7
1.3.14 SPR move . . . 7
1.4 Inference Algorithm . . . 7
1.4.1 Firing probability derivations . . . 8

2 Synthetic data experiments **10**

3 Competitor methods runtime analysis **17**

4 High-quality figures **20**

1 Method details

In this section, we first explain the details of the ToMExO building blocks. In section 1.1, we present our dynamic programming algorithm for estimating the error parameters. We repeatedly use this algorithm in the iterations of our inference procedure. In section 1.2, we provide the details of a gradient descent approach for estimating the error parameters. We use this procedure to fine-tune our error parameters after deciding on the progression model. We provide detailed explanations of our structural moves in section 1.3. Finally, in section 1.4, we explain our inference algorithm in detail.

1.1 Empirical Error Estimation

Given the dataset B and a progression model $T = \big(V, E, \{f_v\}_{v\in V}, \{D_v\}_{v\in V}, P\big)$, we want to calculate an estimation for the error parameters ϵ and δ. We denote the minimum number of false positives and false negatives in $B_{m,v}$ in case of $s_{m,v} = s \in \{0,1\}$ as $Q_{m,v,s} = (n^{\text{FP}}_{m,v,s}, n^{\text{FN}}_{m,v,s})$. We have

$$Q_{m,v,0} = (o_{m,v}, 0), \tag{1}$$

$$Q_{m,v,1} = \begin{cases} (0,1) & , o_{m,v} = 0 \\ (o_{m,v} - 1, 0) & , o_{m,v} \geq 1 \end{cases}. \tag{2}$$

Denoting the minimum number of false positives and false negatives in $B_{m,v\downarrow}$, given $s_{m,v} = s$ as $W_{m,v,s}$, we have

$$W_{m,v,0} = Q_{m,v,0} + \sum_{c\in\mathcal{C}(v)} W_{m,c,0}, \tag{3}$$

$$W_{m,v,1} = Q_{m,v,1} + \sum_{c\in\mathcal{C}(v)} \min_{\|W\|_1}\{W_{m,c,0}, W_{m,c,1}\}. \tag{4}$$

We can calculate these W variables traversing the tree in a post-order manner. Our empirical estimation of the number of false positives and false negatives in the dataset will be:

$$\Big(n_{FP}, n_{FN}\Big) = \sum_{m\in 1,\ldots,M} \big(W_{m,\text{root},1} + (o_{m,P}, 0)\big). \tag{5}$$

Denoting the total number of ones and zeros in the dataset by $\mathcal{O}$ and $\mathcal{Z}$, respectively, we have:

$$\Big(\hat{\epsilon}, \hat{\delta}\Big) = \Bigg(\frac{n_{FP}}{\mathcal{Z} - n_{FN} + n_{FP}}, \frac{n_{FN}}{\mathcal{O} - n_{FP} + n_{FN}}\Bigg). \tag{6}$$

Only a single post-order traversal over the tree is needed to estimate the error parameters. A pseudo-code for this procedure is provided in Algorithm 1.

1.2 Gradient Descent for Error Estimation

As another approach for error parameter estimation, we can use Gradient Descent to find an at least locally optimal estimate of the parameters ϵ and δ, given a progression model T and the dataset

Algorithm 1. Empirical error estimation

Input: B, $T = \big(V, E, \{f_v\}_{v\in V}, \{D_v\}_{v\in V}, P\big)$
Output: $\hat{\epsilon}, \hat{\delta}$
for all $m \in \{1,\ldots,M\}$ **do**
  for $v \in$ post-order(V) **do**
    $o_{m,v} = \|B_{m,D_v}\|_1$
    $Q_{m,v,0} = (o_{m,v}, 0)$
    if $o_{m,v} = 0$ **then**
      $Q_{m,v,1} = (0,1)$
    else
      $Q_{m,v,1} = (o_{m,v} - 1, 0)$
    $W_{m,v,0} = Q_{m,v,0} + \sum_{c\in\mathcal{C}(v)} W_{m,c,0}$
    $W_{m,v,1} = Q_{m,v,1} + \sum_{c\in\mathcal{C}(v)} \min_{\|W\|_1}\{W_{m,c,0}, W_{m,c,1}\}$
$\Big(n_{FP}, n_{FN}\Big) = \sum_{m\in 1,\ldots,M} \big(W_{m,\text{root},1} + (\|B_{m,P}\|_1, 0)\big)$
$\Big(\hat{\epsilon}, \hat{\delta}\Big) = \left(\frac{n_{FP}}{\|1-B\|_1 - n_{FN} + n_{FP}}, \frac{n_{FN}}{\|B\|_1 - n_{FP} + n_{FN}}\right)$

B. Instead of working with the likelihood itself, our objective is to maximize the log-likelihood, which can be written as

$$l(B|T,\epsilon,\delta) = \sum_{m\in\{1,\ldots,M\}} \log(p(B_{m,:}|T,\epsilon,\delta)). \tag{7}$$

We have

$$\frac{\partial l(B|T,\epsilon,\delta)}{\partial\epsilon} = \sum_{m\in\{1,\ldots,M\}} \frac{\partial \log p(B_{m,:}|T,\epsilon,\delta)}{\partial\epsilon}, \tag{8}$$

where

$$\frac{\partial \log p(B_{m,:}|T,\epsilon,\delta)}{\partial\epsilon} = \frac{\partial \log \Psi_{m,\text{root}}}{\partial\epsilon} + \frac{o_{m,P}}{\epsilon} - \frac{z_{m,P}}{1-\epsilon}. \tag{9}$$

We can compute the derivatives of the Ψ variables at the root node with a single post-order traversal of the driver tree. Following the formula used for likelihood calculations, we have

$$\begin{aligned}\frac{\partial \log \Lambda_{m,v}}{\partial\epsilon} = \frac{1}{\Lambda_{m,v}}\Bigg(&\frac{\sum_{g\in D_v: B_{m,g}=1}\alpha_g}{\sum_{g\in D_v}\alpha_g}(1-\delta)\epsilon^{o_{m,v}-2}(1-\epsilon)^{z_{m,v}-1}\big(\epsilon(1-o_{m,v}-z_{m,v}) + o_{m,v} - 1\big)\\ &+ \frac{\sum_{g\in D_v: B_{m,g}=0}\alpha_g}{\sum_{g\in D_v}\alpha_g}\delta\epsilon^{o_{m,v}-1}(1-\epsilon)^{z_{m,v}-2}\big(o_{m,v} - \epsilon(o_{m,v} + z_{m,v} - 1)\big)\Bigg)\end{aligned} \tag{10}$$

$$\frac{\partial \log \Omega_{m,v}}{\partial\epsilon} = \frac{o_{m,v}}{\epsilon} - \frac{z_{m,v}}{1-\epsilon} + \sum_{c\in\mathcal{C}(v)} \frac{\partial \log \Omega_{m,v}}{\partial\epsilon} \tag{11}$$

$$\frac{\partial \log \Psi_{m,v}}{\partial\epsilon} = \frac{\partial \log \Lambda_{m,v}}{\partial\epsilon} + \sum_{c\in\mathcal{C}(v)} \frac{f_c\Psi_{m,c}\frac{\partial \log\Psi_{m,c}}{\partial\epsilon} + (1-f_c)\Omega_{m,c}\frac{\partial\log\Omega_{m,c}}{\partial\epsilon}}{f_c\Psi_{m,c} + (1-f_c)\Omega_{m,c}} \tag{12}$$

Similarly, for the δ variable, we have

$$\frac{\partial l(B|T,\epsilon,\delta)}{\partial\delta} = \sum_{m\in\{1,\dots,M\}} \frac{\partial \log p(B_{m,:}|T,\epsilon,\delta)}{\partial\delta}, \tag{13}$$

where

$$\frac{\partial \log p(B_{m,:}|T,\epsilon,\delta)}{\partial\delta} = \frac{\partial \log \Psi_{m,\mathrm{root}}}{\partial\delta}. \tag{14}$$

To compute the derivatives of the Ψ variables, we have

$$\frac{\partial \log \Lambda_{m,v}}{\partial\delta} = \frac{\epsilon^{o_{m,v}-1}(1-\epsilon)^{z_{m,v}-1}}{\Lambda_{m,v}} \left(\frac{\epsilon\big(\sum_{g\in D_v:B_{m,g}=0}\alpha_g\big) - (1-\epsilon)\big(\sum_{g\in D_v:B_{m,g}=1}\alpha_g\big)}{\sum_{g\in D_v}\alpha_g} \right) \tag{15}$$

$$\frac{\partial \log \Omega_{m,v}}{\partial\delta} = \sum_{c\in\mathcal{C}(v)} \frac{\partial \log \Omega_{m,v}}{\partial\delta} \tag{16}$$

$$\frac{\partial \log \Psi_{m,v}}{\partial\delta} = \frac{\partial \log \Lambda_{m,v}}{\partial\delta} + \sum_{c\in\mathcal{C}(v)} \frac{f_c\Psi_{m,c}\frac{\partial \log \Psi_{m,c}}{\partial\delta} + (1-f_c)\Omega_{m,c}\frac{\partial \log \Omega_{m,c}}{\partial\delta}}{f_c\Psi_{m,c} + (1-f_c)\Omega_{m,c}} \tag{17}$$

Having the derivatives, we can use any gradient-based optimization method to find ϵ and δ.

Note that while we can compute the gradients using a single traverse of the driver tree, the gradient descent methods are iterative processes themselves. Hence, this approach's computational complexity is much higher than the procedure of obtaining empirical error estimates. As the overall performance of our method does not degrade much by using the empirical error estimation, we use the gradient descent algorithm only for fine-tuning the error parameters ϵ and δ after finding the final progression model.

1.3 Structural Moves

To sample progression models using a Metropolis-Hasting approach, we need to have structural moves involving changes in the topology of the driver tree and the assignment of the genes to the nodes. We have designed 14 types of moves to propose new progression models, which may then be accepted based on the Metropolis-Hasting acceptance criteria.

The moves are introduced one by one in the following. Note that whenever we need to *select* one or a set of nodes or genes, we do it uniformly from the set of candidate choices. As an illustrative example, let the progression model shown in Fig. 1-A be the model in hand, i.e., the chain's current state. Fig. 1-B to O show example proposed models for each one of our move types.

1.3.1 Vertical merge

For this move, we merge a leaf node into its parent. We select the leaf node from the leaves that are not in the first layer of the tree so that the resulting tree has a standard structure with no genes in the root node. Fig. 1-B shows an example of such moves.

1.3.2 Vertical split

For this move, we choose a node with at least two genes, select a proper non-empty subset of its genes and move them into a new child node. Fig. 1-C shows an example of such moves.

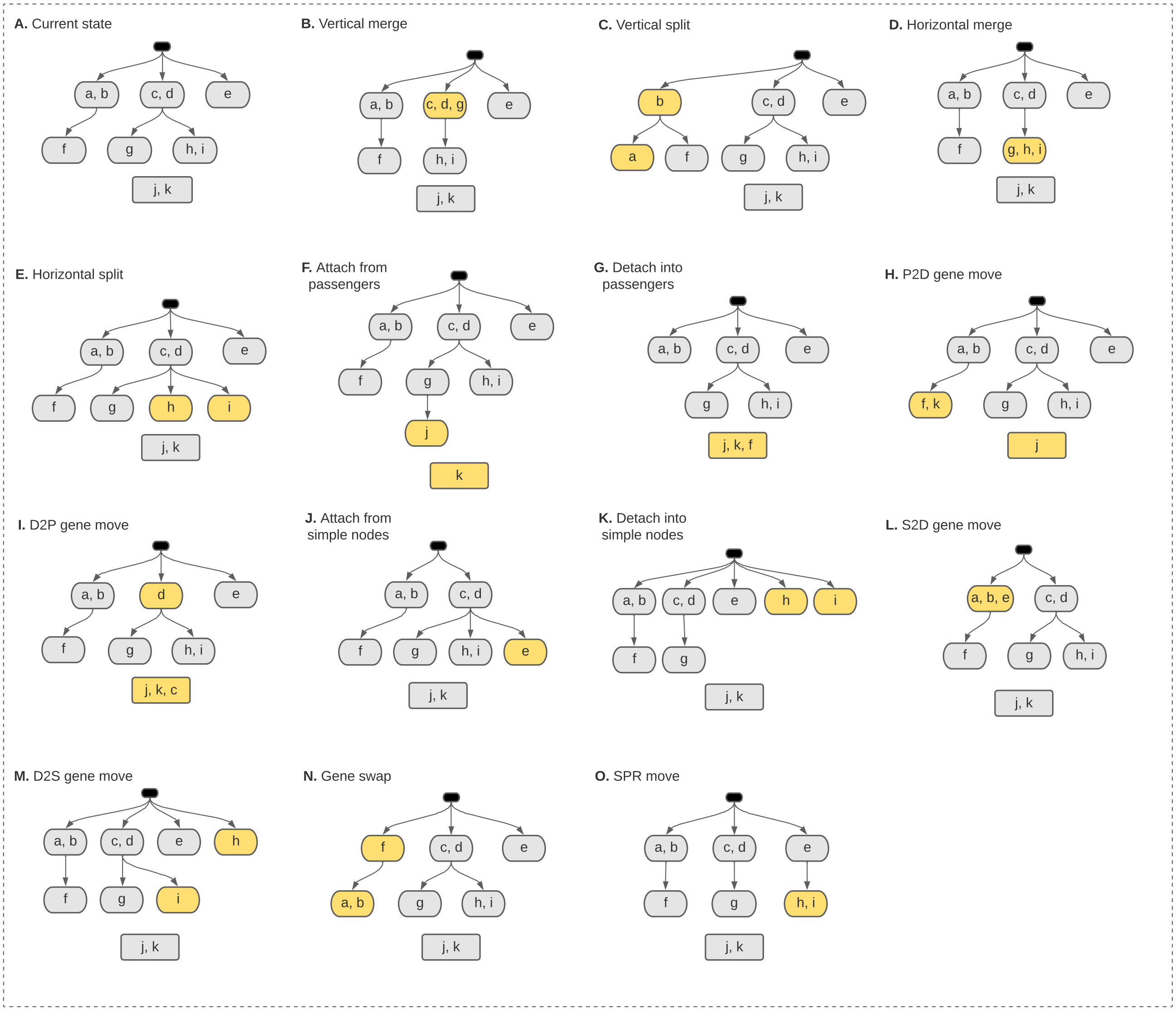

Figure 1: Different types of structural moves used in our MCMC inference algorithm.

1.3.3 Horizontal merge

For this move, we merge a pair of sibling leaves together. Fig. 1-D shows an example of such moves.

1.3.4 Horizontal split

For this move, we split a leaf node into two sibling leaves. To this end, we select a leaf node that includes at least two genes. We partition the selected node's genes into two non-empty subsets and put them into the resulting sibling nodes. Fig. 1-E shows an example of such moves.

1.3.5 Attach from Passengers

For this move, we select a non-empty subset of the passenger genes, form a new node with them, and select its parent node from the existing nodes of the tree. Fig. 1-F shows an example of such

moves.

1.3.6 Detach into Passengers

For this move, we select a leaf node, remove it and put its genes into the set of passengers. Fig. 1-G shows an example of such moves.

1.3.7 P2D gene move

This is the passenger-to-driver gene move. For this move, we select a gene from the set of passengers and move it to one of the nodes in the driver tree. Fig. 1-H shows an example of such moves.

1.3.8 D2P gene move

This is the driver-to-passenger gene move. For this move, we select a node with at least two genes in it, pick one of its genes, and move it to the set of passengers. Fig. 1-I shows an example of such moves.

1.3.9 Attach from simple nodes

With this move, we select a non-empty subset of simple nodes to remove and put their genes in a new node. We attach this new node to a parent node, which

- is not among the selected subset of simple nodes,
- is not the root, if the selected subset includes one or two simple nodes (as these cases will correspond to "no move" and "horizontal merge", respectively).

Fig. 1-J shows an example of such moves.

1.3.10 Detach into simple nodes

With this move, we remove a leaf node and put its genes into new simple nodes. We choose a leaf node, which

- is not a simple node, as it would lead to no move
- is not a first layer node with exactly two genes, as this case is covered in "horizontal split".

Fig. 1-K shows an example of such moves.

1.3.11 S2D gene move

This is the simple-to-driver gene move. For this move, we select a simple node, remove it and put its genes in a node, which

- is not the root node,
- is not a first-level node with no children (to not conflict with "horizontal merge").

Fig. 1-L shows an example of such moves.

1.3.12 D2S gene move

This is the driver-to-simple gene move. For this move, we select a node in the driver tree, which

- has at least two genes
- is not a first-level node with no children (D2S type of moves for such nodes are covered in "horizontal split").

We then select one of the genes in the selected node and move it to a new first-level node, forming a simple node. Fig. 1-M shows an example of such moves.

1.3.13 Gene swap

For this move, we select a node and swap its genes with its parent. We choose our node from those not in the first layer, as the parent node should not be the empty root node. Fig. 1-N shows an example of such moves. It is worth mentioning that the forward and backward probabilities are equal for this move, making it easier to compute the acceptance ratio.

1.3.14 SPR move

This is the well-known subtree pruning and regrafting move. For this move, we select a node, which

- is not the root,
- is not a simple node.

We then select a new parent for the selected node that

- is not in the selected node's subtree (including the selected node itself),
- is not the root node, if the selected node includes one gene and has no children (as it would result in a new simple node).

Note that the simple nodes are the single-gene leaves in the first layer of the tree. As the "attach from simple nodes" covers the SPR moves that relocate the simple nodes, we do not move them here. Note that the SPR move has equal forward and backward probabilities, which is helpful in calculating the acceptance ratio. Fig. 1-O shows an example of such moves.

1.4 Inference Algorithm

Building on our dynamic programming procedure for likelihood calculation (see Algorithm 2), various MCMC approaches can be used for inference. We use the algorithm described in Algorithm 3, where the firing probability of each edge (u, v), denoted by f_v is set to a specific empirical estimation, as

$$f_v = \max\left\{\frac{\mathcal{Y}_{B,v} - \epsilon\mathcal{X}_{B,u}}{(1-\epsilon-\delta)\mathcal{X}_{B,u}}, 0\right\}, \tag{18}$$

Algorithm 2. Likelihood calculation

Input: B, $T = \big(V, E, \{f_v\}_{v\in V}, \{D_v\}_{v\in V}, P\big)$, ϵ, δ
Output: $p(B|T, \epsilon, \delta)$

for all $m \in \{1, \ldots, M\}$ **do** ▷ Calculate $p(B_{m,:}|T, \epsilon, \delta)$
  for $v \in$ post-order(V) **do**
    $o_{m,v} = \|B_{m,D_v}\|_1$
    $z_{m,v} = \|1 - B_{m,D_v}\|_1$
    $\Lambda_{m,v} = \frac{o_{m,v}}{o_{m,v}+z_{m,v}}(1-\delta)\epsilon^{o_{m,v}-1}(1-\epsilon)^{z_{m,v}} + \frac{z_{m,v}}{o_{m,v}+z_{m,v}}\delta\epsilon^{o_{m,v}}(1-\epsilon)^{z_{m,v}-1}$
    $\Gamma_{m,v} = \epsilon^{o_{m,v}}(1-\epsilon)^{z_{m,v}}$
    $\Psi_{m,v} = \Lambda_v \prod_{c\in\mathcal{C}(v)} \big(f_c\Psi_c + (1-f_c)\Omega_c\big)$
    $\Omega_{m,v} = \Gamma_v \prod_{c\in\mathcal{C}(v)} \Omega_c$
  $p(B_{m,:}|T, \epsilon, \delta) = \Psi_{m,\text{root}}\epsilon^{\|B_{m,P}\|_1}(1-\epsilon)^{\|1-B_{m,P}\|_1}$
$p(B|T, \epsilon, \delta) = \prod_{m\in\{1,\ldots,M\}} p(B_{m,:}|T, \epsilon, \delta)$

where

$$\mathcal{X}_{B,u} = \sum_{m\in\{1,\ldots,M\}} \mathbb{1}_{\{\exists g\in D_u | B_{m,g}=1\}}, \tag{19}$$

$$\mathcal{Y}_{B,v} = \sum_{m\in\{1,\ldots,M\}} \mathbb{1}_{\{\exists g\in D_v | B_{m,g}=1\}} * \mathbb{1}_{\{\exists g'\in D_u | B_{m,g'}=1\}}. \tag{20}$$

Note that $\mathcal{X}_{B,u}$ estimates the number of tumors with a mutation in u (the parent node), and $\mathcal{Y}_{B,v}$ estimates the number of tumors with mutations in both v and u. In the following, we prove that the firing probability in (18) is the optimal value for a special case, where the optimal value can be calculated analytically.

1.4.1 Firing probability derivations

Consider a driver tree with only one gene, which has to be placed in a so-called *simple node*. The likelihood of individual tumors can take only two values depending on whether our only gene g (in node v) is mutated or not:

$$p(B_{m,g}|T, \delta, \epsilon) = \begin{cases} f_v(1-\delta) + (1-f_v)\epsilon, & B_{m,g} = 1 \\ f_v\delta + (1-f_v)(1-\epsilon), & B_{m,g} = 0 \end{cases} \tag{21}$$

Taking the derivative of the log-likelihood of the individual tumors with respect to f_v, we have:

$$\frac{\partial \log p(B_{m,g}|T, \delta, \epsilon)}{\partial f_v} = \begin{cases} \frac{1-\delta-\epsilon}{f_v(1-\delta)+(1-f_v)\epsilon}, & B_{m,g} = 1 \\ \frac{\delta+\epsilon-1}{f_v\delta+(1-f_v)(1-\epsilon)}, & B_{m,g} = 0 \end{cases} \tag{22}$$

Summing over all the tumors, the derivative of the total log-likelihood can be written as

$$\frac{\partial \log p(B|T, \delta, \epsilon)}{\partial f_v} = (1-\delta-\epsilon)\bigg(\frac{\sum_m \mathbb{1}_{\{B_{m,g}=1\}}}{f_v(1-\delta) + (1-f_v)\epsilon} - \frac{\sum_m \mathbb{1}_{\{B_{m,g}=0\}}}{f_v\delta + (1-f_v)(1-\epsilon)}\bigg) \tag{23}$$

It can be easily checked that for $\epsilon + \delta < 1$, the second derivative will always be negative, i.e.,

$$\frac{\partial^2 \log p(B|T, \delta, \epsilon)}{\partial f_v^2} < 0. \tag{24}$$

Algorithm 3. ToMExO inference algorithm

Input: B, n_{chains}, $n_{\text{iterations}}$
Output: $T = \big(V, E, \{f_v\}_{v\in V}, \{D_v\}_{v\in V}, P\big)$, ϵ, δ

for all $c \in \{1, ..., n_{\text{chains}}\}$ **do**
  Initialize T_0^c to a star-tree ▷ Using Eq. (18) for $\{f_v\}_{v\in V(T_0^c)}$
  Calculate ϵ_0^c and δ_0^c using Algorithm 1 ▷ Empirical error estimation
  for all $i \in \{1, ..., n_{\text{iterations}}\}$ **do**
    Choose a move type uniformly
    Generate a proposal sample $\hat{T}_i^c$
    Calculate $\hat{\epsilon}_i^c$ and $\hat{\delta}_i^c$ using Algorithm 1 ▷ Empirical error estimation
    Calculate acceptance ratio $p_{i,\text{accept}}^c$ using Eq. (26)
    Draw $q \sim \text{Uniform}(0, 1)$
    if $q < p_{i,\text{accept}}^c$ **then**
      $T_i^c = \hat{T}_i^c$, $\epsilon_i^c = \hat{\epsilon}_i^c$, $\delta_i^c = \hat{\delta}_i^c$ ▷ Accepted proposal
    else
      $T_i^c = T_{i-1}^c$, $\epsilon_i^c = \epsilon_{i-1}^c$, $\delta_i^c = \delta_{i-1}^c$ ▷ Rejected proposal
Choose the best sample $(T, \epsilon, \delta) = \arg\max_{(T_i^c, \epsilon_i^c, \delta_i^c)} p(B|T_i^c, \epsilon_i^c, \delta_i^c)$
Optimize ϵ and δ values using gradient descent (section 1.2)

Setting the derivative equal to zero (and limiting f_v to $[0, 1]$) leads to the formula in (18). Note that in this case of simple nodes, we have

$$\mathcal{X}_{B,u} = M,$$
$$\mathcal{Y}_{B,v} = \sum_m \mathbb{1}_{\{B_{m,g}=1\}}. \tag{25}$$

In our inference algorithm, we use our empirical error estimation (Algorithm 1) instead of sampling the error parameters ϵ and δ. We use a Metropolis-Hasting framework, where in each iteration, we choose a move type and generate a proposal progression model accordingly. We then use Algorithm 1 to calculate the corresponding error parameters ϵ and δ, which form the proposed sample together with the progression model. The proposed sample may then be accepted as the next chain state with a certain probability. Let T_{i-1}^c, ϵ_{i-1}^c and δ_{i-1}^c denote the progression model and error parameters in chain c and iteration $i-1$. Denoting the proposed sample by $(\hat{T}_i^c, \hat{\epsilon}_i^c, \hat{\delta}_i^c)$, the acceptance probability is calculated as

$$p_{i,\text{accept}}^c = \frac{p(\hat{T}_i^c, \hat{\epsilon}_i^c, \hat{\delta}_i^c|B)}{p(T_{i-1}^c, \epsilon_{i-1}^c, \delta_{i-1}^c|B)} * \frac{p_{i,\text{backward}}^c}{p_{i,\text{forward}}^c}, \tag{26}$$

where $p_{i,\text{forward}}^c$ and $p_{i,\text{backward}}^c$ are the forward and backward probabilities.

At the end of the iterations, we pick the sample with the maximum posterior and fine-tune the parameters ϵ and δ using gradient descent, as detailed in section 1.2. A pseudo-code of our inference algorithm is provided in Algorithm 3.

2 Synthetic data experiments

This section provides further details on the results of our synthetic data experiments. As the paper explains, we have three generative progression models shown in Fig. 4 of the paper. For each case, we 16 parameter settings with error rates in $\{0.001, 0.01, 0.05, 0.1\}$ and the number of tumors in $\{50, 100, 200, 500\}$. We generated 10 datasets for each setting and used ToMExO inference algorithm with 1 chain and $100k$ samples to find the maximum a posteriori progression model. Note that while we use the same values for the probability of false-positive ϵ and false-negative δ errors, the inference algorithm views them as independent variables.

Fig. 2 shows the averaged precision and recall values for identifying the mutual exclusivity and progression relations. The resulting F-scores are shown in Fig. 3a and Fig. 3b. Fig. 3c shows our overall scores F_{overall} for the synthetic data experiments. These figures show that our inference algorithm provides excellent results when the error probabilities are up to 0.05, especially when the number of tumors is 100 or more. For most cases with error probability 0.1, our inference algorithm tends to stick to the star tree or a progression model implying very few progression and mutual exclusivity relations, resulting in very low F-scores. For some other cases, when we have only 50 tumors in the data, some relations in deeper levels of the progression model are difficult to identify, leading to low F-scores. Consider the linear model with $\epsilon = 0.05$, for example. As shown in Fig. 2, when we have only 50 tumors, the precision and recall values are unsatisfactory. Notably, the recall values for progression and mutual exclusivity relations are way lower than their corresponding precision values. The problem in this example case is resolved as the number of tumors in the data increases.

As another way of measuring our performance, we used two distance metrics introduced in [2], originally designed for clonal trees. Consider a gene g in a progression model T. Let the *ancestral set* of g in T, $A_T(g)$, be the set of genes in the same node as g or its ancestor nodes. We define the *common ancestor set* $C_T(g, w)$ as $A_T(g) \cap A_T(w)$. We also define the *distinctly inherited set* to be $D_T(i, j) = A_T(g) \setminus A_T(w)$. The Common Ancestor Set distance (CASET) between two progression models T and $\hat{T}$ is defined as:

$$\text{CASET}(T, \hat{T}) \triangleq \frac{1}{\binom{|G|}{2}} \sum_{\{g,w\} \subseteq G} \text{Jacc}(C_T(g, w), C_{\hat{T}}(g, w)), \tag{27}$$

where G is the set of genes and *Jacc* is the *Jaccard distance*, i.e.,

$$\text{Jacc}(A, B) = \frac{|A \cup B| - |A \cap B|}{|A \cup B|}. \tag{28}$$

The Distinctly Inherited Set distance (DISC) between two progression models T and $\hat{T}$ is defined as:

$$\text{DISC}(T, \hat{T}) \triangleq \frac{1}{|G|(|G| - 1)} \sum_{(g,w) \in G^2 : g \neq w} \text{Jacc}(D_T(g, w), D_{\hat{T}}(g, w)). \tag{29}$$

Note that both DISC and CASET are in $[0, 1]$, where DISC or CASET being 0 implies the two progression models are identical. Fig. 4 shows our results distances to the generative models. As shown in this figure, the DISC and CASET distances follow the same pattern as our F-scores in a smoother way.

To better explain the results, Fig 5a shows the averaged posterior ratios of the star tree to the

generative progression models, normalized by the number of tumors:

$$\left(\frac{p(T_0, \epsilon_0, \delta_0|B)}{p(T_g, \epsilon_g, \delta_g|B)}\right)^{1/M}, \tag{30}$$

where $(T_0, \epsilon_0, \delta_0)$ denotes the star tree and it's corresponding error values, and $(T_g, \epsilon_g, \delta_g)$ are the generative tree and error variables. As shown in the figure, when the error rates increase, the generative model gradually loses its advantage over the star tree, and the star tree gets a likelihood very close to the generative model. As a result, it is more difficult for the inference algorithm to improve from the initial state, which is the star tree, as it already provides a very competitive likelihood. Fig 5b shows the normalized posterior ratios of the output trees to the generative progression models. This figure shows that for almost all the cases with error probabilities up to 0.05, we have found a model with a likelihood at least as good as the generative model. As a sanity check, we can see that in all 48 cases, the output gives a posterior ratio at least as high as the initial star tree.

The averaged fine-tuned error parameters provided in the outputs are shown in Fig. 6a and Fig. 6b. As expected, these figures show that the estimated error parameters are typically lower than the generative values. The figures also show a general trend of increasing inferred values as the generative error parameter increases. In some seemingly strange cases, the inferred ϵ and δ values are very small, while the generative error probability equals 0.1. See the case of the linear model with 500 tumors and $\epsilon = 0.1$ as an example. The reason for such poor performance in estimating the error parameters is that the output trees are the star trees or very similar. Having the star tree as the output, we do not need a high error probability to get a good likelihood, mostly because of the perfectly matching firing probabilities.

Fig. 7 shows the averaged per-iteration run-time of ToMExO (in seconds). This figure shows that the run-time increases (sub)linearly with the number of tumors. Moreover, the generative model has almost no impact on the algorithm's run-time. Note that increment in the noise level barely affects the run-time, as expected.

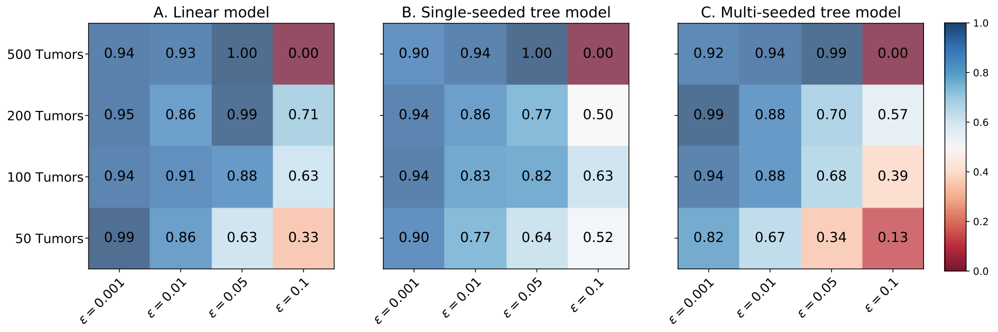

(a) Progression precision

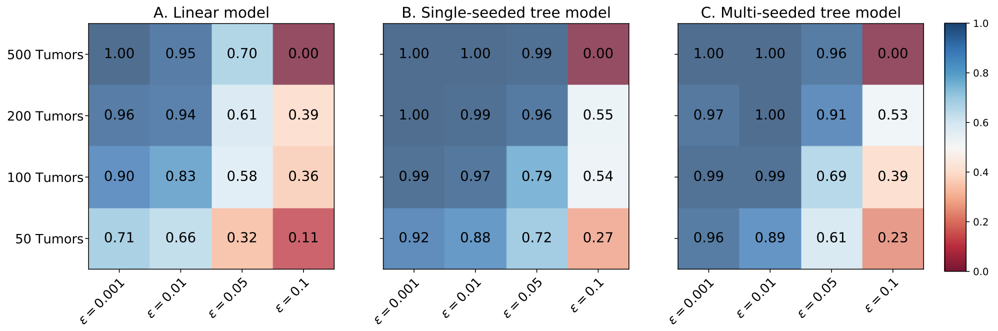

(b) Progression recall

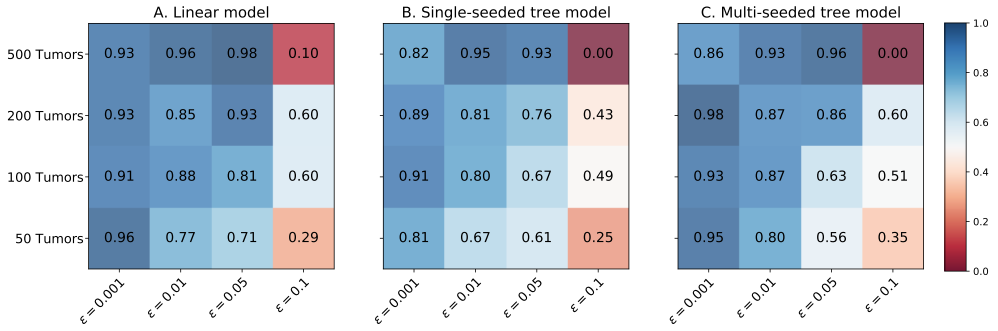

(c) Mutual exclusivity precision

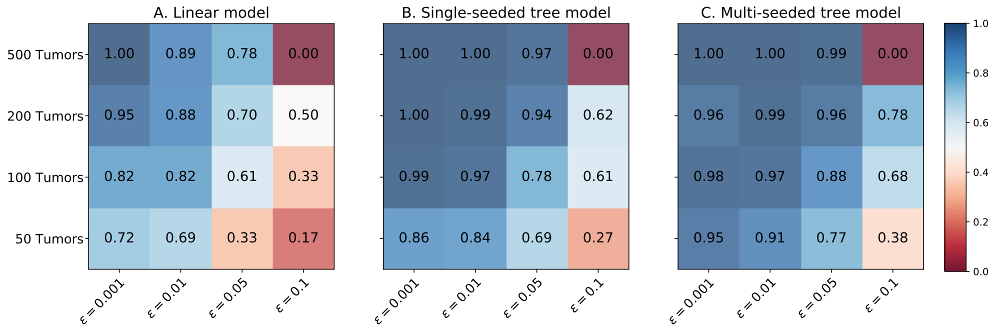

(d) Mutual exclusivity recall

Figure 2: The precision and recall values (averaged over 10 datasets)

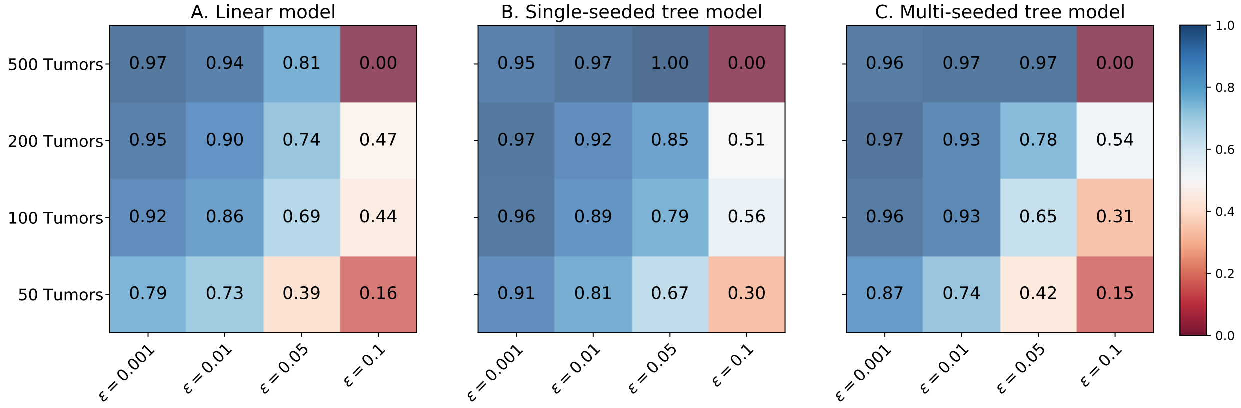

(a) Progression F-scores, F_{PR}

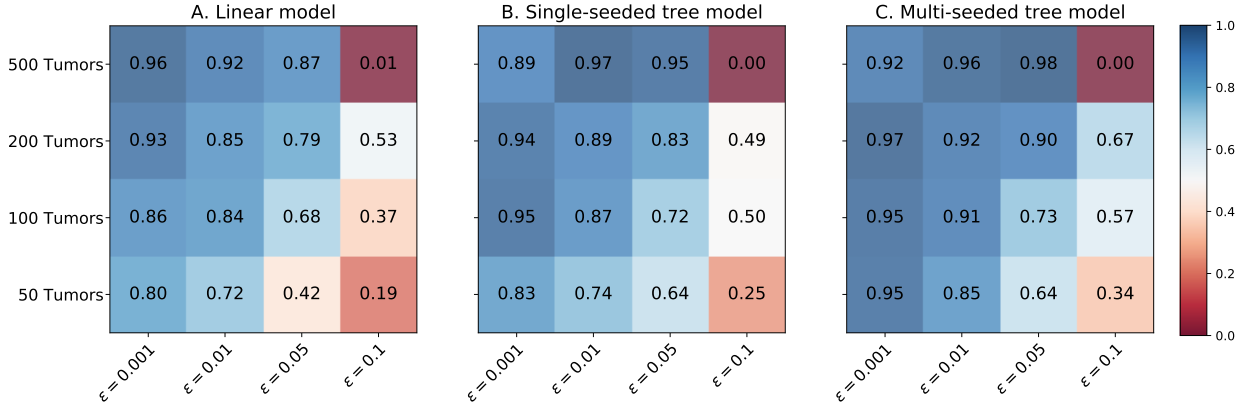

(b) Mutual exclusivity F-scores, F_{ME}

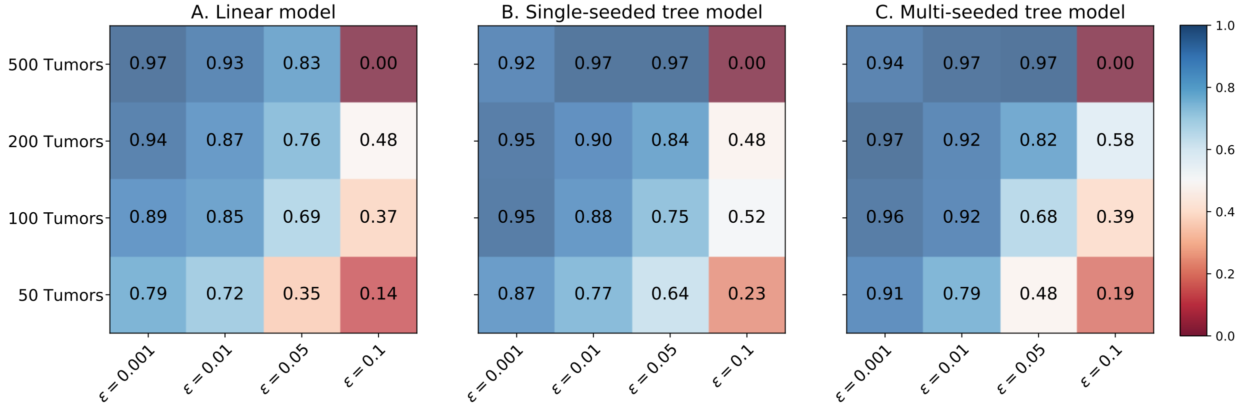

(c) Overall F-scores, F_{overall}

Figure 3: The F-scores (averaged over 10 datasets)

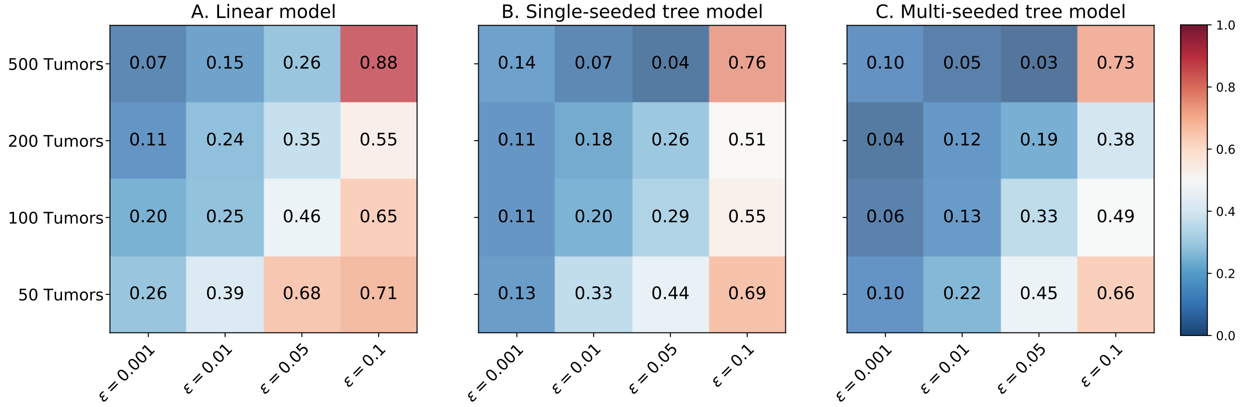

(a) DISC distance

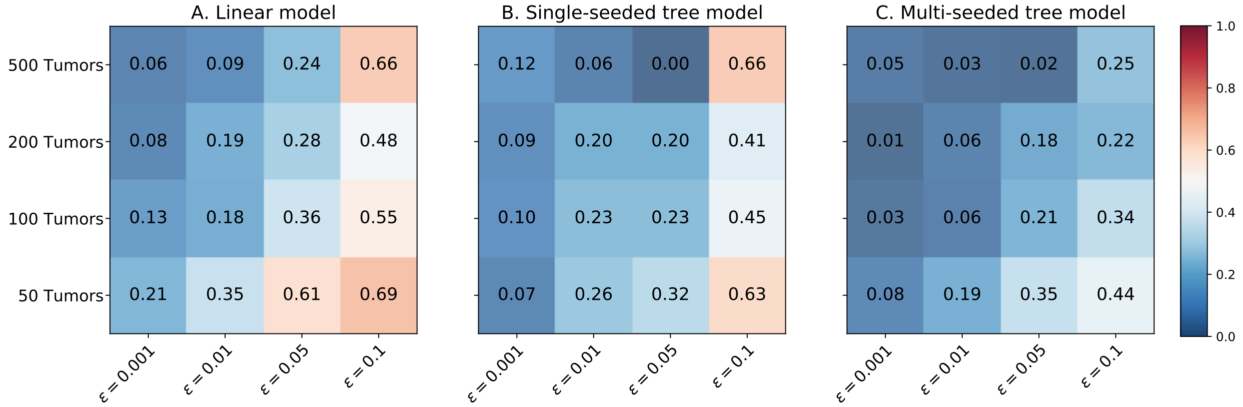

(b) CASET distance

Figure 4: Averaged distance between the results and the generative models

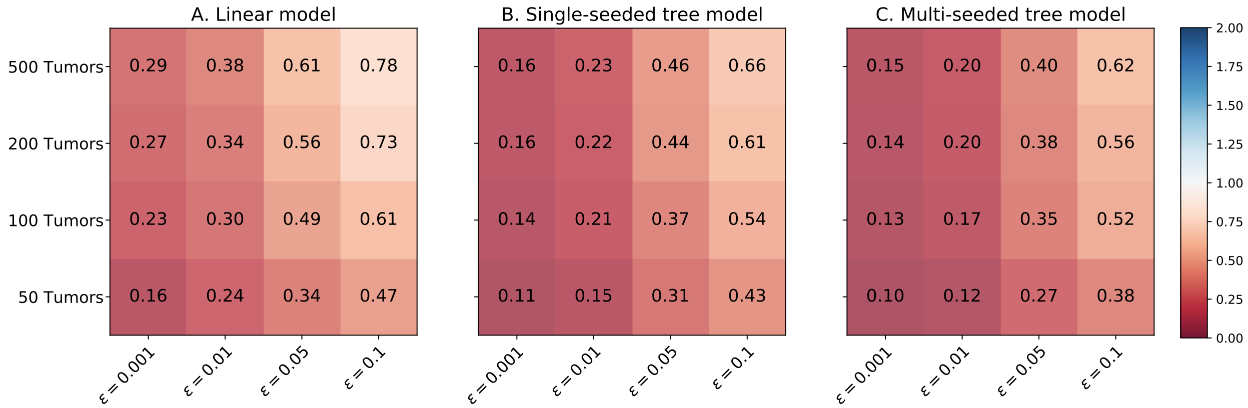

(a) The averaged ratio of the star tree to the generative model posteriors (normalized for the number of tumors)

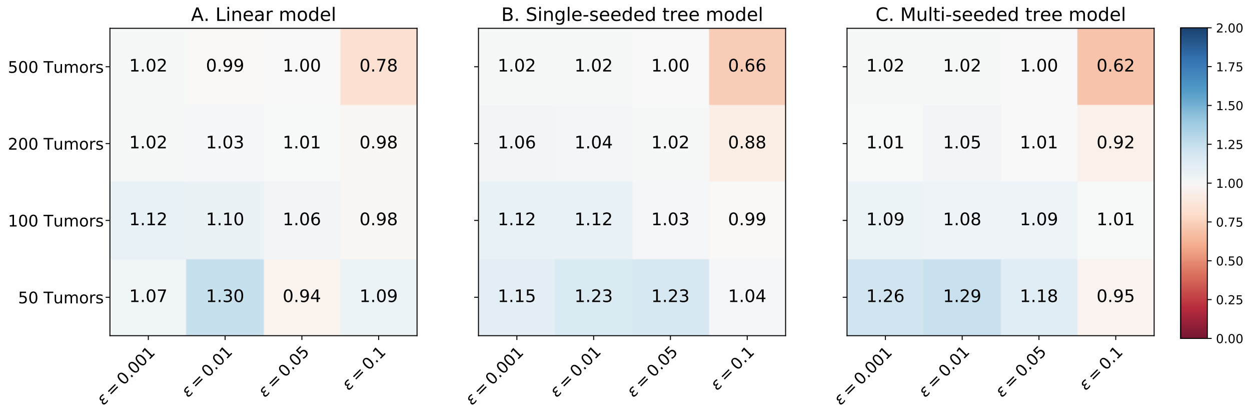

(b) The averaged ratio of the output tree to the generative model posteriors (normalized for the number of tumors)

Figure 5: The posterior ratios

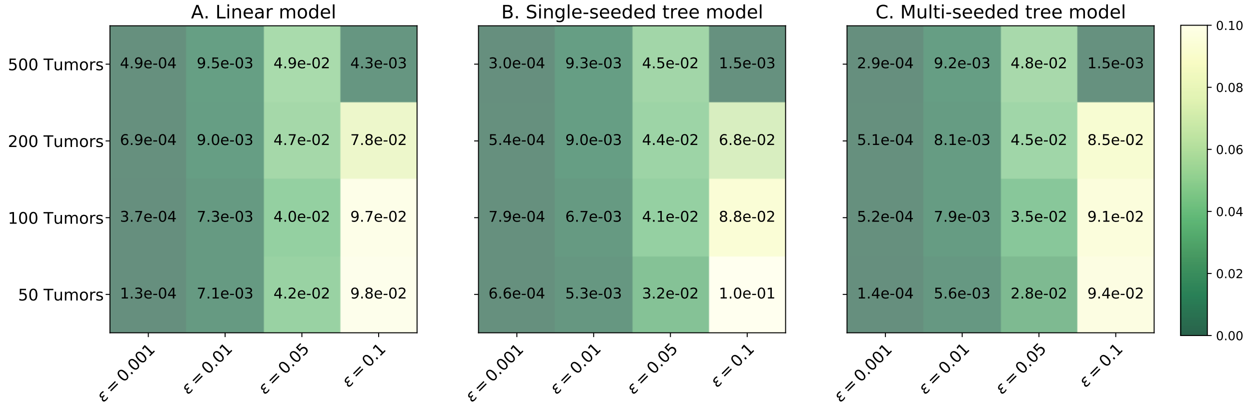

(a) Probability of false positive, ϵ

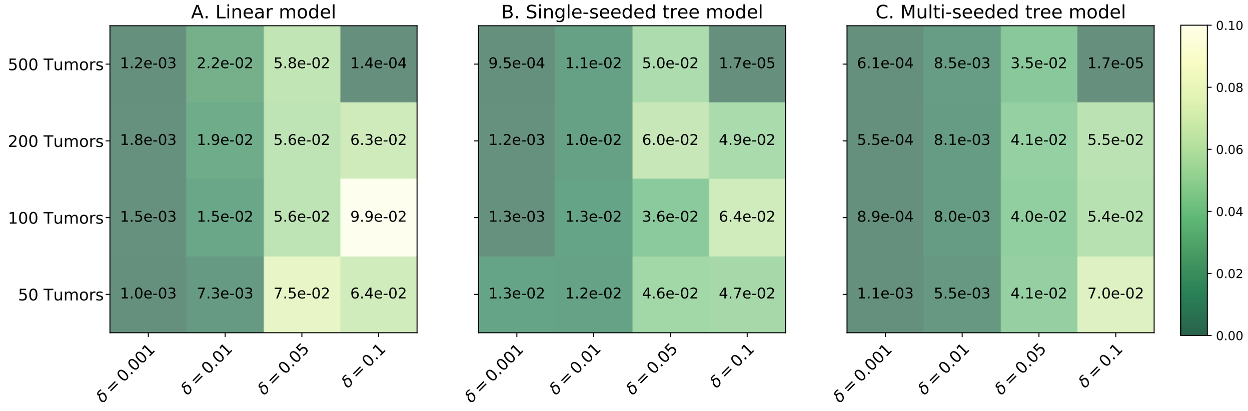

(b) Probability of false negative, δ

Figure 6: The averaged inferred error parameters

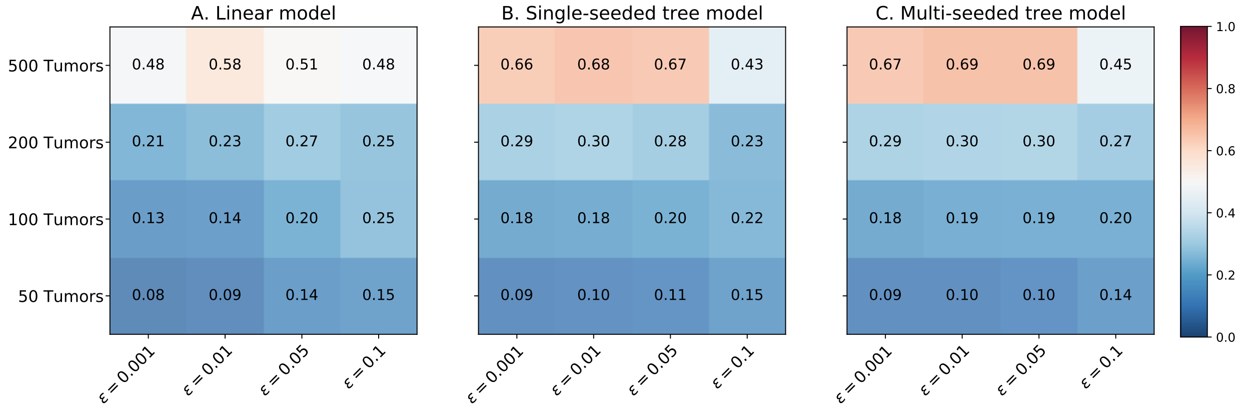

Figure 7: The per-iteration run-time of ToMExO (in seconds), averaged over 10 datasets per case.

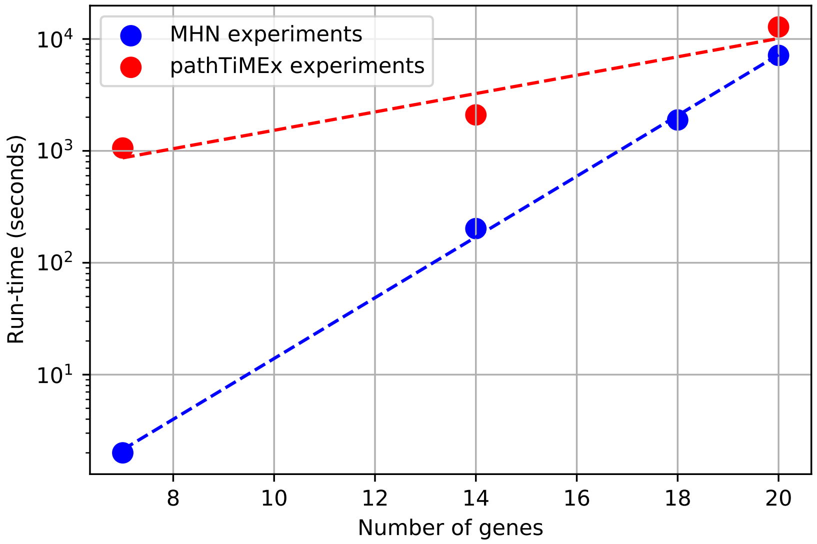

Figure 8: The run-time of pathTiMEx and MHN methods with respect to the number of genes in the dataset.

3 Competitor methods runtime analysis

As explained in the paper, the competitor methods, pathTiMEx [1] and MHN [3], have severe problems with a high number of genes in the data. Fig. 9 shows the exact errors we got in a set of experiments on datasets of various sizes and the run-time for the experiments that could be run. For both competitors, we used the implementations published with their corresponding papers:

- For pathTiMEx: https://github.com/cbg-ethz/pathTiMEx
- For MHN: https://github.com/RudiSchill/MHN

Fig. 8 shows the exponential computational complexity of both pathTiMEx and MHN with respect to the number of genes (note the logarithmic Y axis). We emphasize that while the computational complexity of MHN should be independent of the number of tumors (as it works with the distribution of mutations in the space of possible genotypes), pathTiMEx computational complexity increases with the number of tumors. Here, due to the limited amount of run-time data, we only focus on showing that the pathTiMEx run-time seems exponential in the number of genes.

For the pathTiMEx experiments, we got a warning regarding "Too many genes to add; please update the list!" after the initialization of the mutually exclusive sets for runs involving TCGA STAD, COADREAD, and UCEC datasets. This warning originates from "funcsTiMEx.R", where the warning is raised if a group of size 20 or more is in the initialization grouping. These three experiments later resulted in an error (See Fig. 9).

For the MHN experiments, we observed an exponential run-time with respect to the number of genes in the successful runs. Moreover, when working on a dataset with N genes, the MHN method needs to construct vectors of length 2^N. This results in a hard threshold on the number of genes the MHN method can handle. As shown in the corresponding error description, we couldn't run MHN on datasets with more than 30 genes (See Fig. 9).

Dataset	Number of genes	Number of tumors	pathtimex runtime	mhn runtime
TCGA KICH	7	66	1061 s	2 s
TCGA CHOL	14	35	2097 s	202 s
TCGA PAAD	18	150	E1 after 2807 s	1889 s
TCGA STAD	32	289	E2 after 9 s	E3 after 4 s
TCGA COADREAD	43	223	E2 after 30 s	E3 after 4 s
TCGA UCEC	53	248	E2 after 25 s	E3 after 4 s
TCGA GBM	26	290	E1 after 58136 s	Halted after 604239 s
pathTiMEx Glioblastoma	20	261	12817 s	7138 s

- **E1:**
```
Error:  Number of events in poset and data do not match!
Error in read.table(paste(completeFilestem, "/paramsEps.lambda", sep = ""),  :
  no lines available in input
Calls: pathTiMEx -> runCBN -> read.table
Execution halted
```

- **E2:**
```
Error in proposedgroupys[[nodetomovefrom]] :
  attempt to select less than one element in get1index <real>
Calls: pathTiMEx -> optGroups
Execution halted
```

- **E3:**
```
Error in tabulate(Data, nbins = N) :
  attempt to make a table with >= 2^31 elements
Calls: Data.to.pD -> tabulate
Execution halted
```

Figure 9: Feasibility and run-time analysis of pathTiMEx and MHN on different datasets. The cells colored in green show the run-times in seconds. The cells colored in red indicate the experiments facing errors.

References

[1] Simona Cristea, Jack Kuipers, and Niko Beerenwinkel. pathtimex: joint inference of mutually exclusive cancer pathways and their progression dynamics. *Journal of Computational Biology*, 24(6):603–615, 2017.

[2] Zach DiNardo, Kiran Tomlinson, Anna Ritz, and Layla Oesper. Distance measures for tumor evolutionary trees. *Bioinformatics*, 36(7):2090–2097, 2020.

[3] Rudolf Schill, Stefan Solbrig, Tilo Wettig, and Rainer Spang. Modelling cancer progression using mutual hazard networks. *Bioinformatics*, 36(1):241–249, 2020.

4 High-quality figures

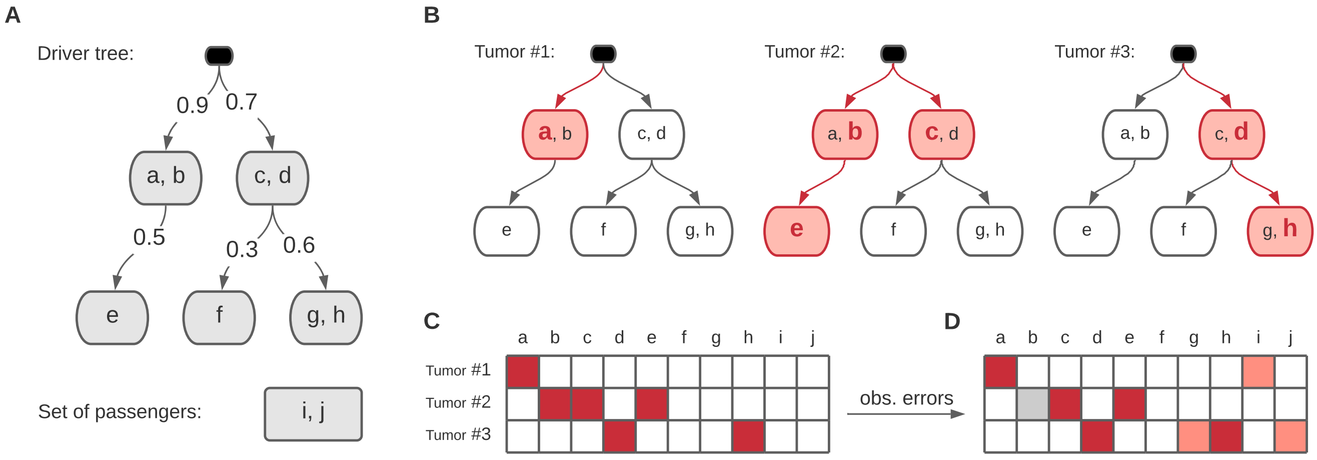

Manuscript Fig. 1

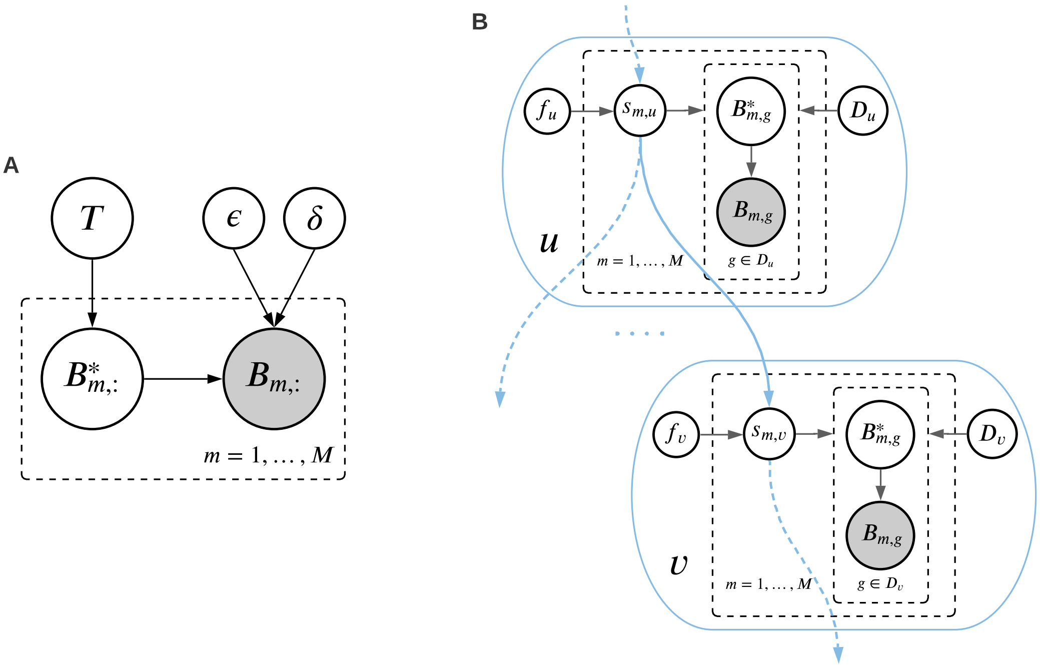

Manuscript Fig. 2

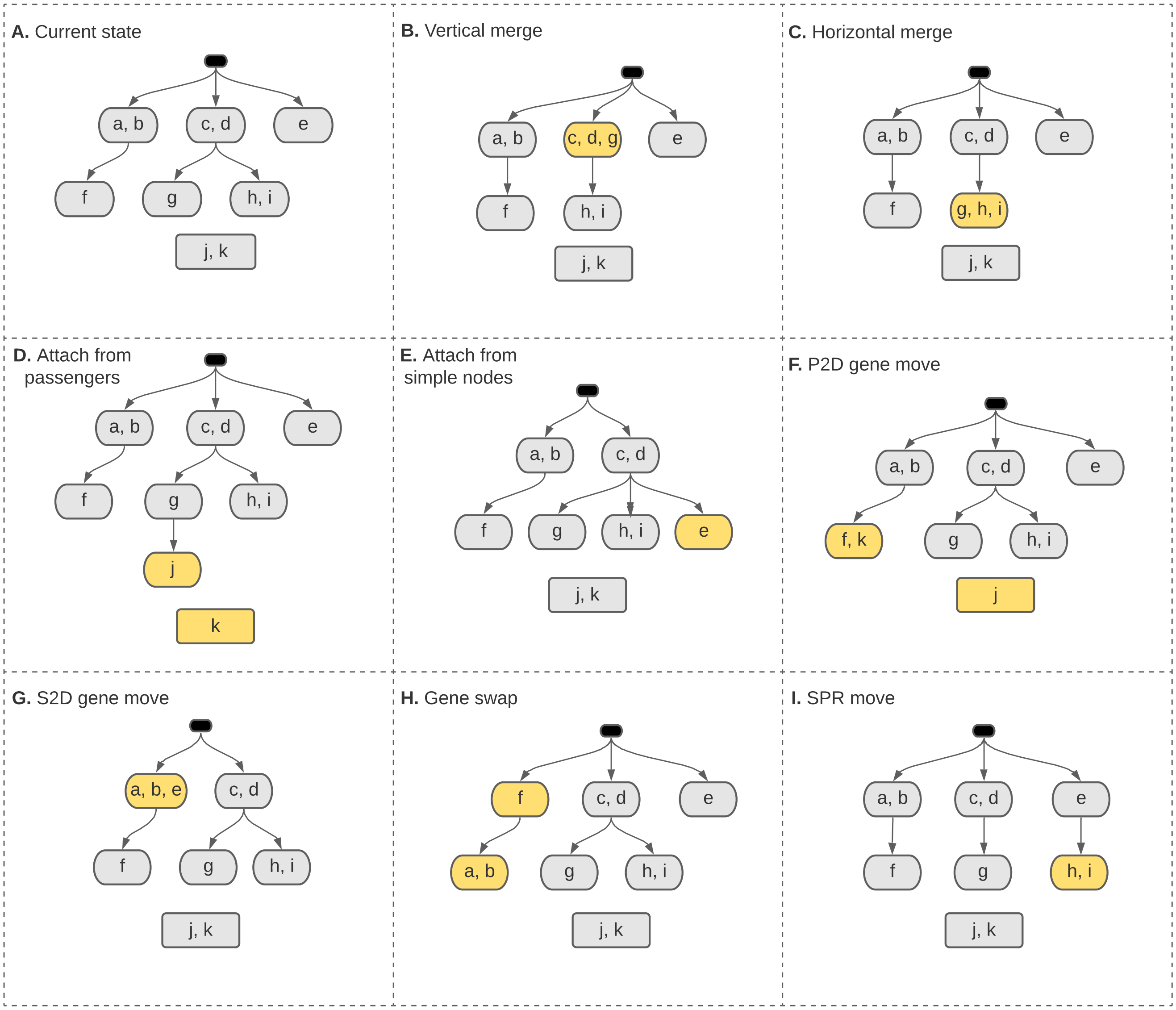

Manuscript Fig. 3

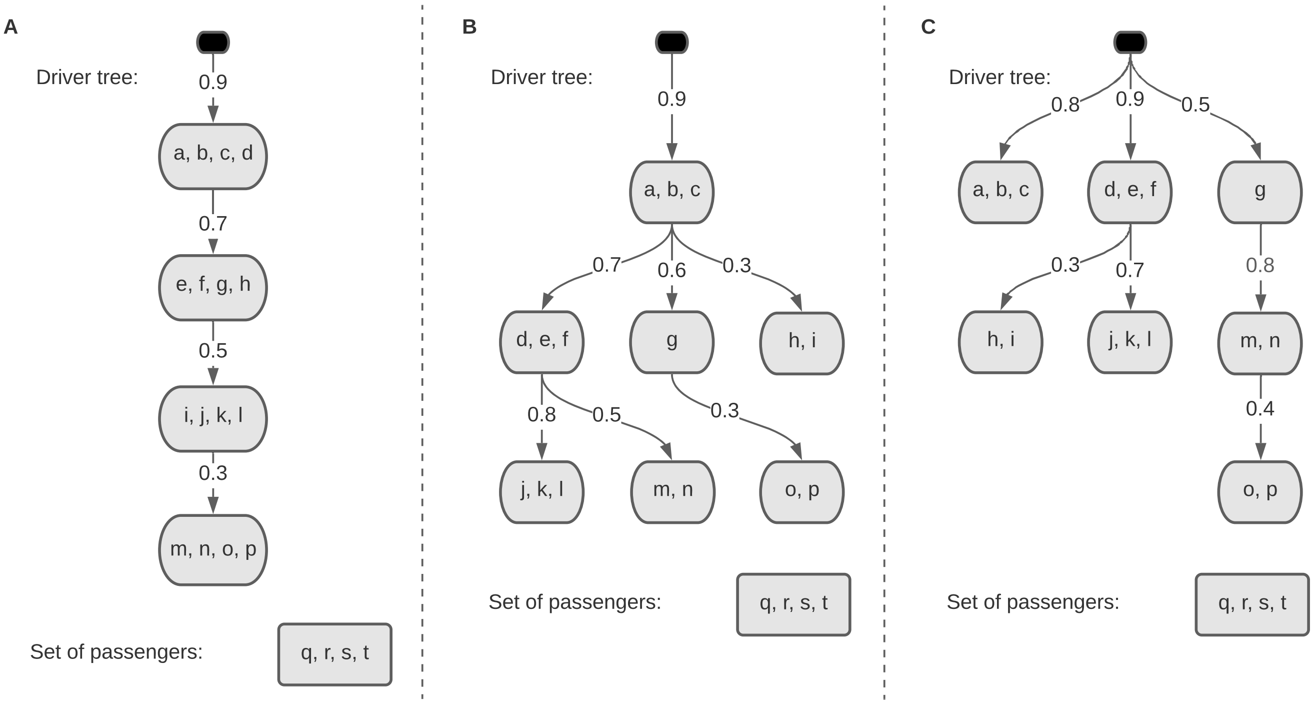

Manuscript Fig. 4

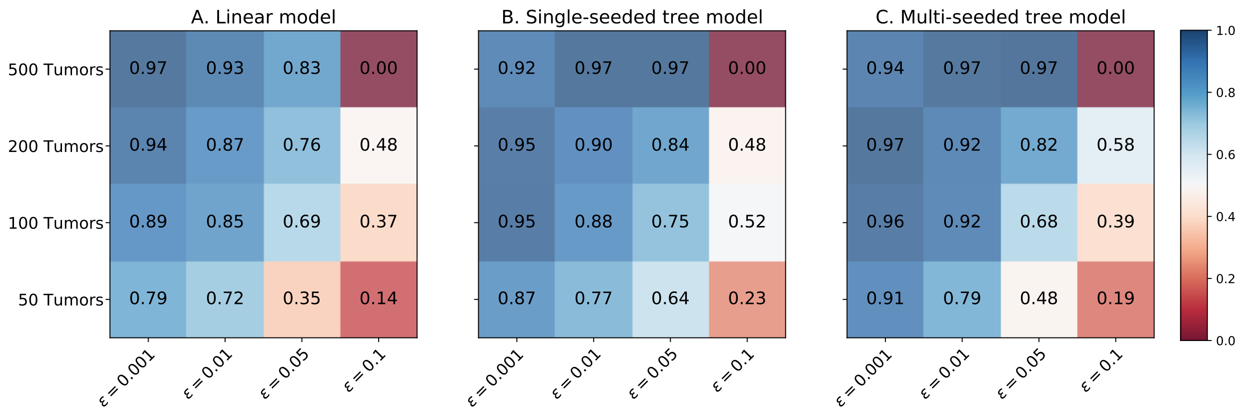

Manuscript Fig. 5

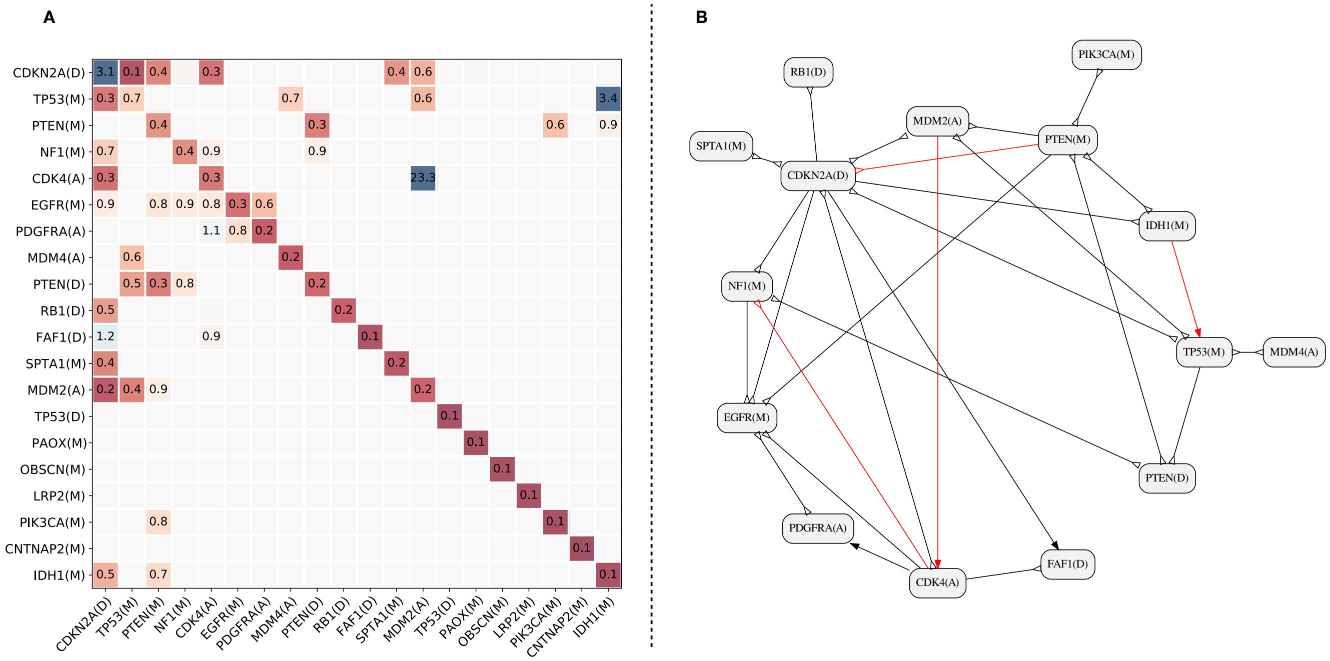

Manuscript Fig. 6

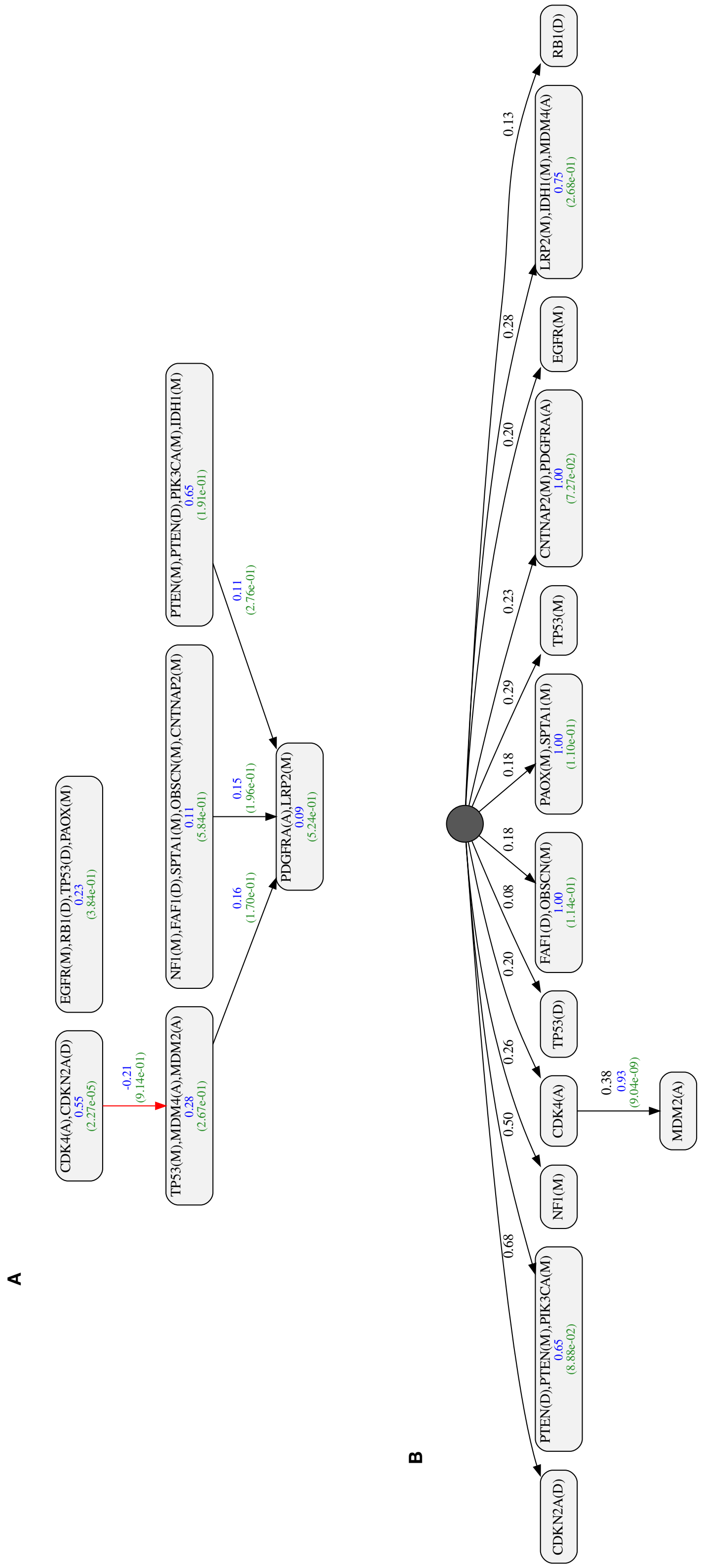

Manuscript Fig. 7

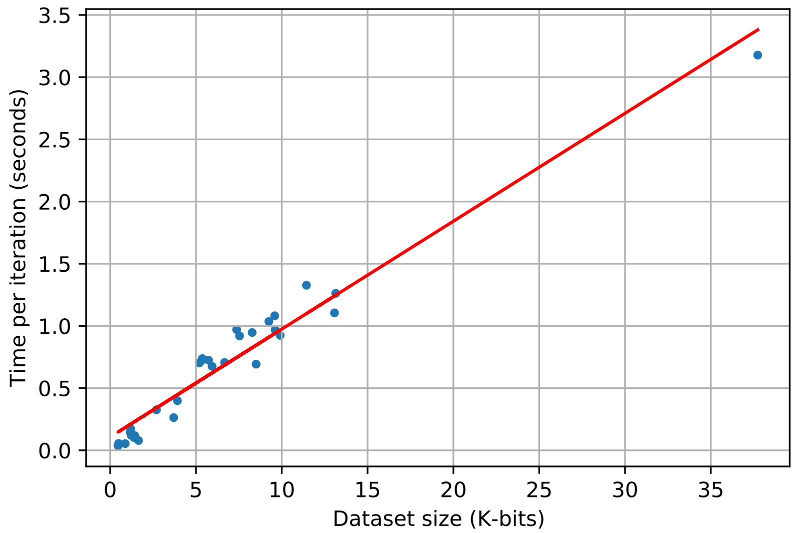

Manuscript Fig. 8

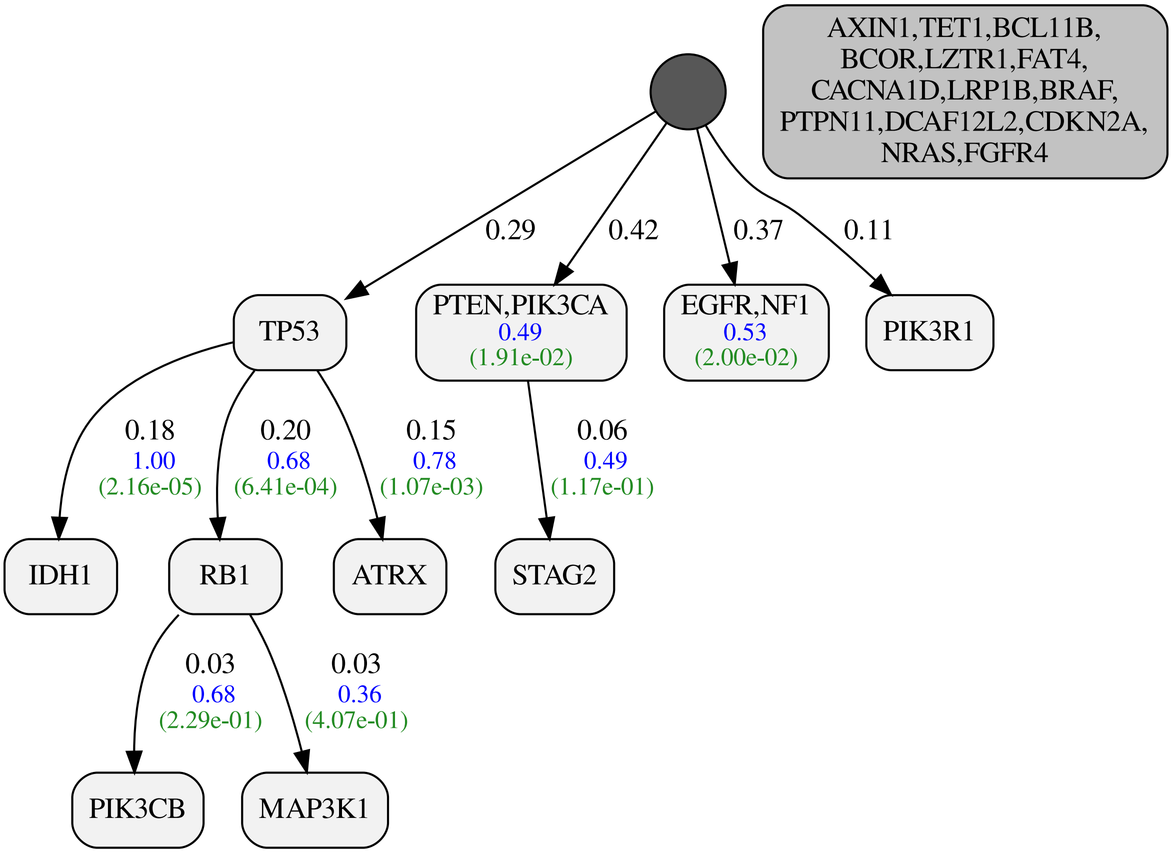

Manuscript Fig. 9

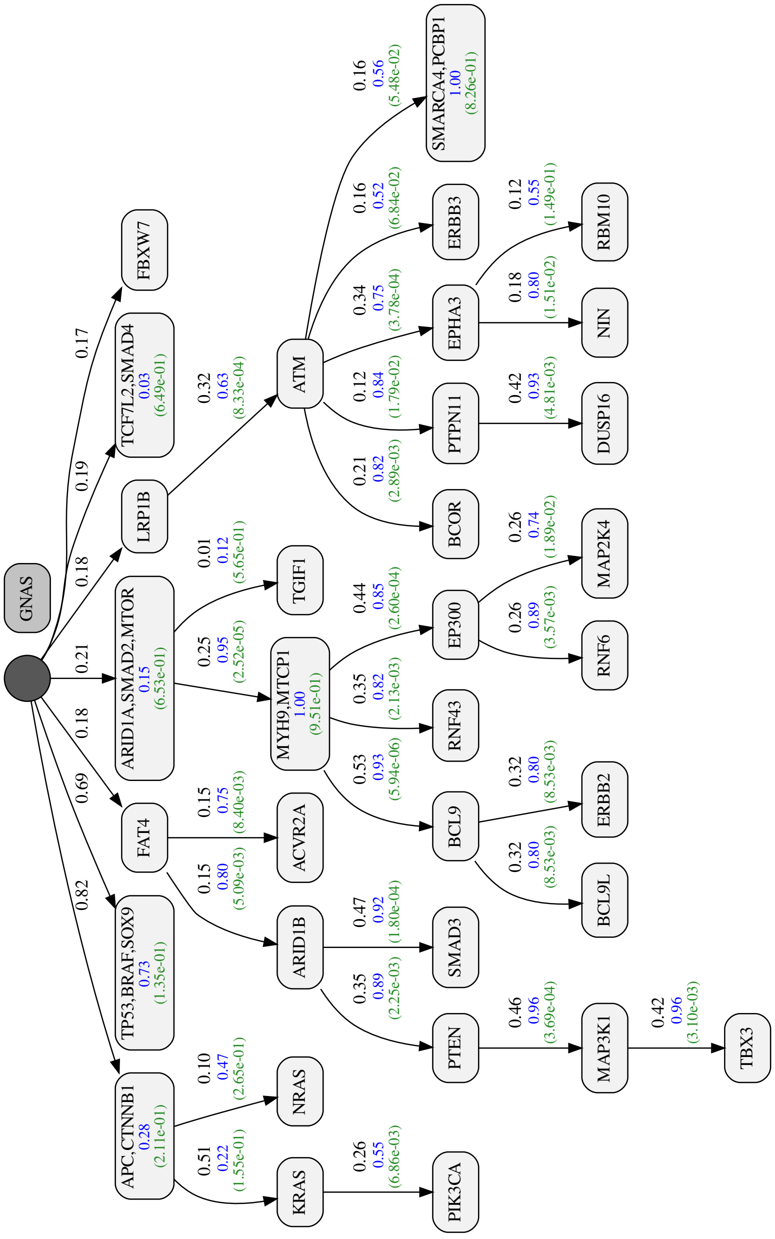

Manuscript Fig. 10

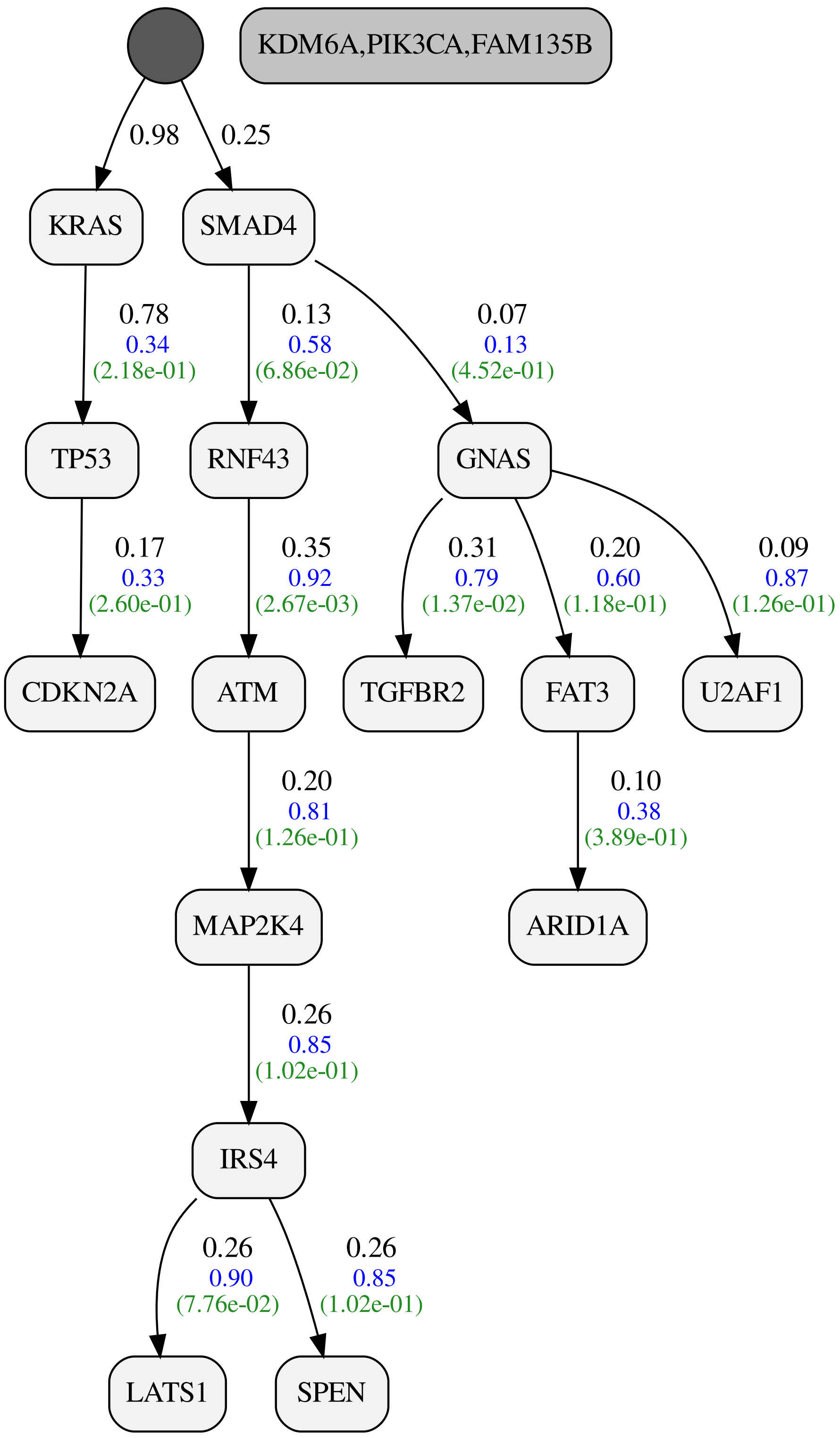

Manuscript Fig. 11
